# Vitamins and Minerals for Blood Pressure Reduction in the General, Normotensive Population: A Systematic Review and Meta-Analysis of Six Supplements

**DOI:** 10.3390/nu15194223

**Published:** 2023-09-30

**Authors:** Benjamin J. Behers, Julian Melchor, Brett M. Behers, Zhuo Meng, Palmer J. Swanson, Hunter I. Paterson, Samuel J. Mendez Araque, Joshua L. Davis, Cameron J. Gerhold, Rushabh S. Shah, Anthony J. Thompson, Binit S. Patel, Roxann W. Mouratidis, Michael J. Sweeney

**Affiliations:** 1 College of Medicine, Florida State University, 1115 W Call Street, Tallahassee, FL 32304, USA; jm15v@med.fsu.edu (J.M.); hip19@med.fsu.edu (H.I.P.); jld21d@med.fsu.edu (J.L.D.); cjg17e@med.fsu.edu (C.J.G.); rss22c@med.fsu.edu (R.S.S.); ajt16b@med.fsu.edu (A.J.T.); roxann.mouratidis@med.fsu.edu (R.W.M.); michael.sweeney@med.fsu.edu (M.J.S.); 2 College of Medicine, University of South Florida, 560 Channel Side Drive MDD 54, Tampa, FL 33602, USA; brettbehers@usf.edu (B.M.B.); mendezaraque@usf.edu (S.J.M.A.); 3 Department of Statistics, Florida State University, 117 N Woodward Ave., Tallahassee, FL 32306, USA; zmeng@fsu.edu (Z.M.); pjswanson@fsu.edu (P.J.S.); 4 Internal Medicine Residency, Florida State University, 1700 South Tamiami Trail, Sarasota, FL 34239, USA; binit-patel@smh.com

**Keywords:** vitamins, minerals, nutraceuticals, blood pressure reduction, normotensive, general population

## Abstract

Hypertension is the leading preventable risk factor for cardiovascular disease and all-cause mortality worldwide. However, studies have shown increased risk of mortality from heart disease and stroke even within the normal blood pressure (BP) range, starting at BPs above 110–115/70–75 mm Hg. Nutraceuticals, such as vitamins and minerals, have been studied extensively for their efficacy in lowering BP and may be of benefit to the general, normotensive population in achieving optimal BP. Our study investigated the effects of six nutraceuticals (Vitamins: C, D, E; Minerals: Calcium, Magnesium, Potassium) on both systolic blood pressure (SBP) and diastolic blood pressure (DBP) in this population. We performed a systematic review and pairwise meta-analysis for all six supplements versus placebo. Calcium and magnesium achieved significant reductions in both SBP and DBP of −1.37/−1.63 mm Hg and −2.79/−1.56 mm Hg, respectively. Vitamin E and potassium only yielded significant reductions in SBP with values of −1.76 mm Hg and −2.10 mm Hg, respectively. Vitamins C and D were not found to significantly lower either SBP or DBP. Future studies should determine optimal dosage and treatment length for these supplements in the general, normotensive population.

## 1. Introduction

Approximately 1.39 billion people in the world have hypertension, the leading preventable risk factor for cardiovascular disease and all-cause mortality worldwide [1]. Hypertension can contribute to and cause both heart disease and stroke, the first and fifth leading causes of death in the United States as of 2017 [2]. Given these statistics, the importance of blood pressure (BP) control is evident. Currently, the American College of Cardiology/American Heart Association (ACC/AHA) defines normal BP as systolic < 120 mm Hg and diastolic < 80 mm Hg, with stage 1 hypertension starting at systolic BP of 130–139 mm Hg or diastolic BP of 80–89 mm Hg [3]. However, prior literature suggests that adverse effects on health can be seen even within the normal BP range. In a meta-analysis of nearly one million people without known vascular disease, a positive correlation was seen between vascular mortality and BPs above 115/75 [4]. This study also suggested that, for the general normotensive population, even consistent reductions of 2 mm Hg in systolic BP could result in large reductions of disabling strokes and premature deaths from vascular causes [4]. Another meta-analysis of 147 randomized controlled trials (RCTs) found that BPs above 110/70 were correlated with increased deaths from coronary heart disease (CHD) and stroke [5]. The authors suggested that some patients, although considered normotensive by conventional definitions, may benefit from treatment with antihypertensives to reduce this increased mortality risk [5].

Aside from prescription antihypertensives, there is ample literature regarding the BP-lowering ability of various nutraceuticals [6,7]. These nutraceuticals include vitamins (C, D, and E) and minerals (calcium, magnesium, and potassium). Studies have addressed both the mechanisms of action and efficacy of these nutraceuticals for BP reduction. Vitamins C and E are thought to mediate BP through antioxidant effects and enhancement of nitric oxide pathways that prevent endothelial dysfunction [8,9,10]. Vitamin D is a regulator of the renin-angiotensin-aldosterone system (RAAS) [11]. The mechanism underlying calcium is not well-understood but is suspected to be regulated by the parathyroid hormone, vitamin D, and RAAS systems [12]. Magnesium can affect BP by inducing vascular changes through the production of nitric oxide, by indirectly affecting intracellular calcium concentrations, and through the alteration of smooth muscle tone [13]. Finally, potassium exhibits a direct effect on BP via two mechanisms: by downregulation of the sodium-chloride cotransporter within the distal tubule of the kidneys leading to reduced reabsorption of sodium and chloride, as well as increasing activation of RAAS in response to high serum potassium levels [14].

The effectiveness of these six nutraceuticals in lowering BP, when taken as dietary supplements, has been the focus of numerous randomized controlled trials (RCTs) and meta-analyses. In fact, the most recently published meta-analyses suggest that all but vitamin D and calcium are capable of achieving a 2 mm Hg systolic BP reduction [15,16,17,18,19,20]. However, the majority of these meta-analyses have included RCTs where the supplements were used as first-line treatments among patients with uncontrolled hypertension. This limits the generalizability of BP reductions for normotensive patients and likely overestimates their true effect. 

This study aims to investigate the effects of the above six nutraceuticals (Vitamins: C, D, E; Minerals: Calcium, Magnesium, Potassium) on systolic and diastolic BP in the general, normotensive population using a pairwise meta-analysis for each supplemental compared to placebo. We chose these six based on our prior knowledge of the literature. The overlap of supplements mentioned in the two aforementioned review articles on nutraceuticals with a blood pressure-lowering effect was also used in this decision [6,7]. Further, we used these review articles to confirm the presence of RCTs examining the effect of supplementation on BP to perform our analysis. We hypothesize finding similar efficacy of these nutraceuticals to what is seen in the most recent published meta-analyses of them. However, we also hypothesize our reductions will be lower than what they obtained because of our controlling for this population, thus excluding studies in which these supplements were used in solely hypertensive patients. To our knowledge, this will be the first study to investigate the effect of all six of these nutraceuticals amongst normotensive subjects in the general population within a single paper. We hope the availability of this data will aid clinicians and patients in determining the efficacy of these nutraceuticals and deciding whether any may be of benefit. Furthermore, it seeks to lay the foundation for future studies that may determine the optimal dosages, treatment length, and long-term safety profiles of these nutraceuticals in this population.

## 2. Methods

This study adhered to the Preferred Reporting Items for Systematic Reviews and Meta-analysis (PRISMA) statement [21]. The protocol was not registered.

### 2.1. Eligibility Criteria

Studies were included if they were (1) published in English; (2) randomized controlled trials (RCTs) of one, or more if not used solely in combination, of the six supplements with dosing versus placebo; (3) focused on a general, adult participant population with <50% having a common medical condition; (4) trials that reported the mean effect with variance of these supplements on systolic, diastolic, or mean arterial pressure (or provided data that allowed for calculation of this data, such as *p*-values and 95% confidence intervals); and (5) at least two weeks in duration. It should be noted that our study did not consider the following as medical conditions: being elderly, postmenopausal, obese, at-risk of a medical condition (such as being prediabetic) or having baseline deficiency of a supplement. Aside from these, all other medical conditions were cause for exclusion if they affected more than 50% of the study population, including hyperlipidemia, alcoholism, smoking, and psychiatric conditions. 

Trials were excluded if (1) they were not published in English; (2) they did not follow a RCT study design; (3) the supplement dosing was not given, was administered via fortified foods or through dietary changes, or was calculated by urinary excretion or other indirect measures; (4) they focused solely on patients with specific medical conditions or had ≥50% of participants sharing a common medical condition; (5) participants were less than 18 years old or pregnant; (6) the average baseline systolic BP was ≥140 or baseline diastolic BP was ≥90; (7) they did not provide data on pre- and post-intervention BP, or sufficient data to calculate this; (8) additional interventions were administered alongside the supplement, such as an exercise program or sodium restriction; and/or (9) the studies were less than two weeks in duration.

### 2.2. Information Sources and Search Strategy

A systematic search of literature was conducted on Cochrane, Embase, MEDLINE (PubMed), and Web of Science for placebo-controlled RCTs of the six supplements examining their effects on BP. The dates searched were inception to 22 July 2022. The search strings were constructed by a medical librarian using a combination of keywords and Medical Subject Headings (MeSH terms). The keywords and MeSH terms used in this search are listed in Table S1 of the Supplementary Data file. Additional studies were retrieved by handsearching the references of all included studies. It should be noted that Vitamin B was included in the literature search but was not used in this study due to the number of different types of B vitamins and their variable mechanisms and effects.

### 2.3. Selection Process

The RCTs obtained through this search were imported into Covidence, which automatically screened for duplicates [22]. Two independent researchers (BJB and JM) screened the title and abstracts of studies, excluding irrelevant ones. The full-text articles were obtained for studies that made it through this initial screening. The same two authors then independently screened these, examining their adherence to eligibility criteria. Disagreements during the screening process were settled through discussion, with a third author (MJS) available to decide any that could not be resolved.

### 2.4. Data Collection Process and Data Items

Two independent researchers (JLD and CJG) completed extraction of the basic characteristics of included studies, with subsequent review by the first author (BJB). This included the last name of the first author, year of publication, type of trial, baseline BP of participants and their past medical history, length of the trial, supplement dosing, sample size for intervention and placebo groups, and whether there were any adverse outcomes. Disagreements were settled through discussion.

The first author (BJB) extracted all data in relation to systolic blood pressure (SBP) and diastolic blood pressure (DBP) for all arms of the trials. This included mean baseline and final BP values for each group, effect sizes for each arm of the trial, and overall treatment effect sizes (supplement minus placebo). The corresponding variances, 95% Confidence Intervals (CIs), and sample sizes for each arm of the trial were also extracted. This was subsequently verified by at least two other authors (BMB, SMA, JLD, or CJG) for accuracy.

### 2.5. Study Risk of Bias Assessment

Quality assessment was performed using the Risk of Bias 2 tool from the Cochrane Handbook for Systematic Reviews of Interventions [23]. This tool examined risk of bias across five domains: (1) Arising from the randomization process; (2) Arising from deviations from the intended interventions; (3) Due to missing outcome data; (4) From the method of measuring the outcome; and (5) In selection of the reported result [23]. Two authors (BJB and RSS) independently performed this, assigning each domain either “Low”, “Some Concerns”, or “High”. Studies with one or more domains receiving “High” grades or more than two domains with “Some Concerns” were considered at overall high risk of bias. Further, studies with two domains with “Some Concerns” were deemed to have some concerns for bias overall. Disagreements were resolved through discussion.

### 2.6. Effect Measures and Statistical Analysis

The primary endpoints were the effect size between the intervention (supplement) and placebo groups in regard to change in systolic blood pressure (SBP) and diastolic blood pressure (DBP). This was represented by the mean BP changes from baseline in the intervention group minus the changes in the placebo group. Its standard error (SE) was either reported or calculated from 95% CIs, *p*-values, and/or t-statistics. When the effect size and its SE were not provided, the effect size was calculated by subtracting the mean changes between the two groups. The SE was then estimated by multiplying the pooled standard deviations of the two groups by the square root of the sum of the reciprocals of their sample sizes. Our effect sizes are the overall effect size of the supplement versus placebo across all dosages and treatment lengths, with the intention of providing insight into whether these nutraceuticals have any effect on BP in this population.

In parallel trials that did not provide mean change from baseline data for each arm of the trial, this was calculated by subtracting the baseline mean BP values from the final mean BP values. The standard deviations of these changes were then imputed according to Chapter 6 of the Cochrane Handbook for Systematic Reviews of Interventions (Cochrane Handbook), assuming a correlation coefficient of 0.7 [24]. A sensitivity analysis adopting a correlation coefficient of 0.5 was also conducted. For studies with multiple treatment arms with different dosages of the supplement or with different subtypes of the same supplement, these arms were combined into one intervention group using the methodology outlined in the Cochrane Handbook. This combined treatment group was then compared against the placebo group in a pairwise analysis. When trials had participants separated into subgroups with both treatment and placebo arms, we treated the subgroups as if they were separate trials. 

For cross-over studies, the final mean BP values of the two groups were used to calculate the mean differences, as all participants shared the same baseline values. Our methodology followed Chapter 23 of the Cochrane Handbook [25]. The SEs were obtained by dividing the standard deviations of the differences by the square root of the sample sizes with an imputed correlation coefficient of 0.7. Sensitivity analyses adopting correlation coefficients of 0.5 and 0.9 were also conducted.

The overall effect sizes and the 95% CIs for each intervention were estimated and reported under both common- and random-effects settings. Heterogeneity was assessed by *Q* statistic at the significance level of 0.1 [26]. A significant *Q* statistic would indicate use of the random-effects model results. The *I*^2^ statistic was also reported to explain the percentage of variability accounted for by the between-study variation [27]. This between-study variance was estimated using the restricted maximum likelihood (REML) method [28]. The publication bias was assessed using Egger’s regression, at a significance level of 0.1 [29]. Contour-enhanced funnel plots of the six supplements with respect to SBP and DBP were also constructed to visualize potential publication bias [30,31]. Statistical analyses were conducted by using R 4.3.0 and the R package ‘meta’ [32].

## 3. Results

### 3.1. Study Selection

**Figure 1:**
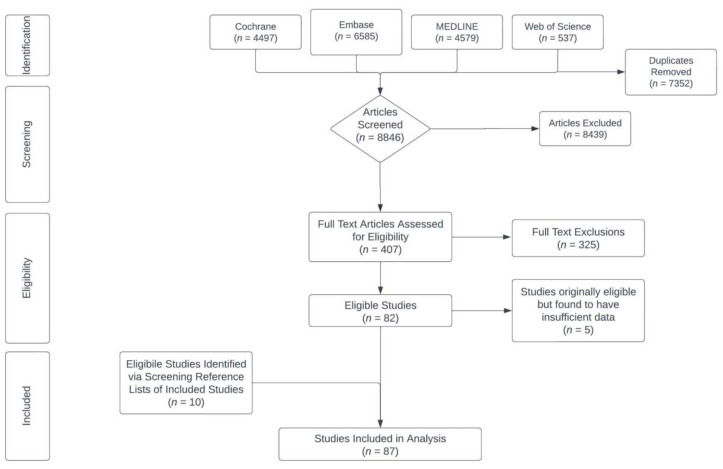
PRISMA flowchart documenting the literature search and screening process.

The literature search yielded 16,198 total articles from the four databases: Cochrane (*n* = 4497), Embase (*n* = 6585), MEDLINE (PubMed) (*n* = 4579), and Web of Science (*n* = 537). Covidence automatically removed 7352 duplicates, leaving 8846 articles for screening. After title/abstract screening removed 8439 articles, full texts were obtained for the remaining 407 articles. Analysis for their adherence to the inclusion criteria resulted in 82 eligible studies [33,34,35,36,37,38,39,40,41,42,43,44,45,46,47,48,49,50,51,52,53,54,55,56,57,58,59,60,61,62,63,64,65,66,67,68,69,70,71,72,73,74,75,76,77,78,79,80,81,82,83,84,85,86,87,88,89,90,91,92,93,94,95,96,97,98,99,100,101,102,103,104,105,106,107,108,109,110,111,112,113,114]. However, five of these were later deemed ineligible due to insufficient data for analysis [68,77,78,80,84]. The full list of reasons for excluded full-text articles and explanations for these five studies can be found in Tables S2 and S3, respectively. Ten additional eligible studies were identified by scanning the reference lists of included studies [115,116,117,118,119,120,121,122,123,124], for a total of 87 RCTs included in the analysis. The study selection process is summarized with a PRISMA flowchart in Figure 1.

### 3.2. Study Characteristics

The 87 RCTs were published from 1982 to 2022 [33,34,35,36,37,38,39,40,41,42,43,44,45,46,47,48,49,50,51,52,53,54,55,56,57,58,59,60,61,62,63,64,65,66,67,69,70,71,72,73,74,75,76,79,81,82,83,85,86,87,88,89,90,91,92,93,94,95,96,97,98,99,100,101,102,103,104,105,106,107,108,109,110,111,112,113,114,115,116,117,118,119,120,121,122,123,124]. The basic characteristics of each included study can be found in Table S4. These 87 RCTs resulted in 95 pairwise comparisons for SBP and 91 for DBP, as some trials had treatment arms for multiple supplements or distinct subgroups with both treatment and placebo groups. Overall, these trials enrolled 12,526 participants. Forty-four of the trials (50.6%) consisted of an entirely healthy population. The remaining trials were broken down into 20 (23.0%) with a general population, 15 trials (17.2%) with healthy but obese participants, and 8 (9.2%) focused on postmenopausal women. There were 71 parallel trials and 16 cross-over. Trial lengths varied from 2 weeks to 208 weeks (4 years).

All six supplements consisted of a mixture of parallel and cross-over studies, except for vitamin D which consisted of only parallel trials. Additionally, all 87 trials (100%) included data on SBP, while DBP was also measured in all but four (95.4%). Of these four trials, two were of vitamin E and one for both vitamin C and vitamin D. A healthy population was predominant for all six supplements. Vitamin C, vitamin D, and vitamin E were represented in 5, 29, and 7 trials, respectively, with sample sizes of 122, 4897, and 302. Calcium, magnesium, and potassium were represented in 21, 18, and 12 trials, respectively, with sample sizes of 4534, 1575, and 1096. As noted, not all studies measured DBP, resulting in slight decreases in the sample size used in its analysis. There was a wide range of dosages and treatment lengths for all six supplements. A comprehensive summary of the basic characteristics of included studies broken down by supplement is depicted in Table 1.

### 3.3. Risk of Bias in Studies

**Figure 2:**
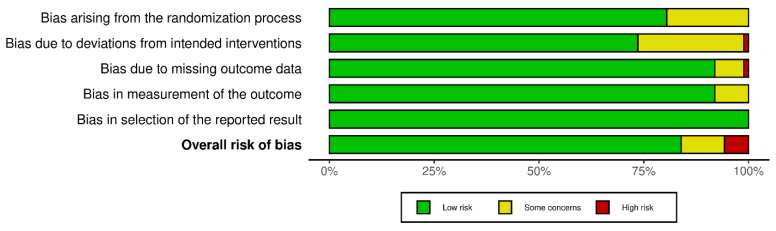
Graphical representation of the risk of bias across all five domains, as well as the overall judgment, for all included studies.

The complete results of the Cochrane Risk of Bias screening by study can be found in Table S5. Overall, five studies (5.7%) were determined to have high risk of bias, while nine (10.3%) were determined to have some concerns. Domain 2, bias arising from deviations from the intended interventions, was the most implicated of the five, with one (1.1%) study having high risk of bias and 22 (25.3%) having some concerns. Domain 1, bias arising from the randomization process, was second with 17 (19.5%) studies having some concerns, but none exhibiting high risk of bias. The Risk of Bias results across the five domains, as well as the overall judgment, for all included studies can be seen in Figure 2.

A summary of the risk of bias judgments by supplement can be found in Table 2. Calcium had the most studies at high risk of bias with 2 (9.5% of its total studies), while vitamin D (3.4% of its total studies), vitamin E (14.3%), and potassium (8.3%) each had one.

### 3.4. Results of Syntheses/Statistical Analyses

#### 3.4.1. Systolic Blood Pressure

**Figure 3:**
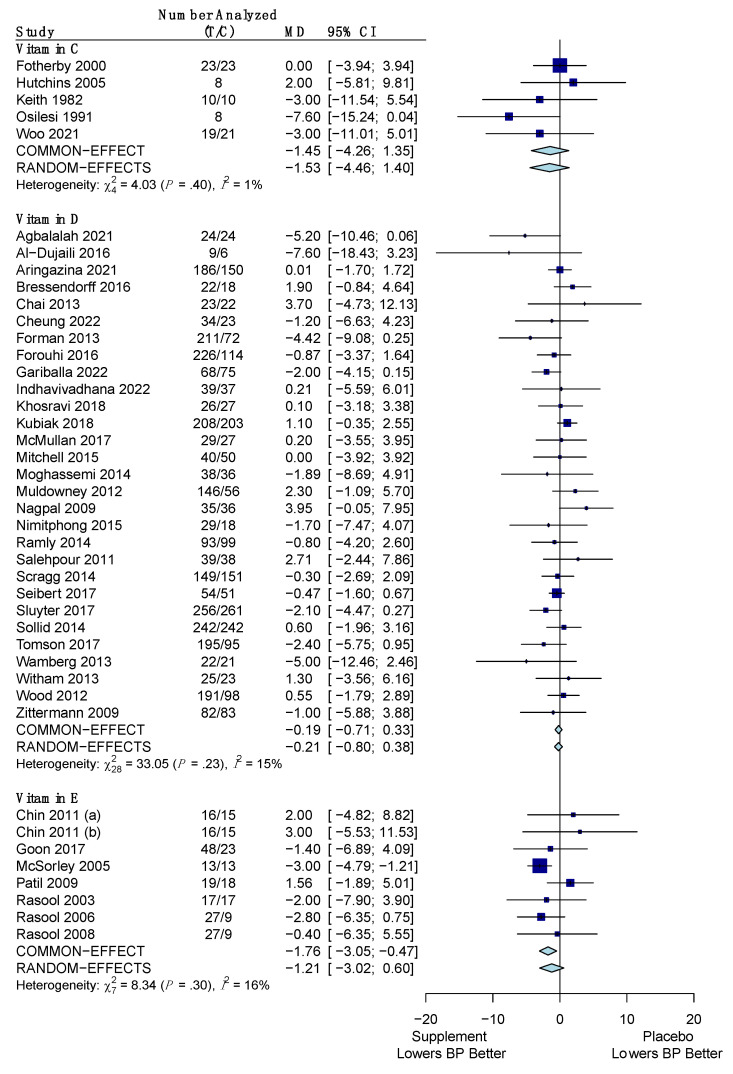
Forest plot showing the mean difference of change in systolic blood pressure between vitamins (C, D, E) and placebo. T represents the sample size of the treatment (supplement/vitamin) group and C represents the sample size of the control (placebo) group. The pooled mean difference is given in mm Hg with its 95% CI. The weighted mean difference in mm Hg and its 95% CI is given for each study. A graphical representation with the weighted mean difference and its SE is also given for each study. References: Vitamin C ([51,56,61,85,112]); Vitamin D ([33,34,35,42,43,44,49,50,52,57,63,64,74,75,79,81,83,87,97,99,100,104,105,107,108,111,112,113,122,124]); Vitamin E ([86,88,89,90,115,117,120]).

The pooled results for the difference in the change of SBP for the vitamins versus placebo are shown in Figure 3. Overall, out of the three vitamins (C, D, E), only vitamin E was found to significantly reduce SBP versus placebo by a mean difference of −1.76 mm Hg (95% CI: −3.05, −0.47) using the common effects model due to low heterogeneity (*I*^2^ = 16%; *p*-value for *Q* statistic: 0.30). Vitamins C and D did not show a significant reduction in SBP versus placebo with values of −1.45 mm Hg (95% CI: −4.26, 1.35) and −0.47 mm Hg (95% CI: −2.29, 1.34), respectively. Both of these vitamins also had low heterogeneity (*I*^2^ = 1%; *p*-value: 0.40 and *I*^2^ = 15%; *p*-value: 0.23, respectively), so a common effects model was used for them.

**Figure 4:**
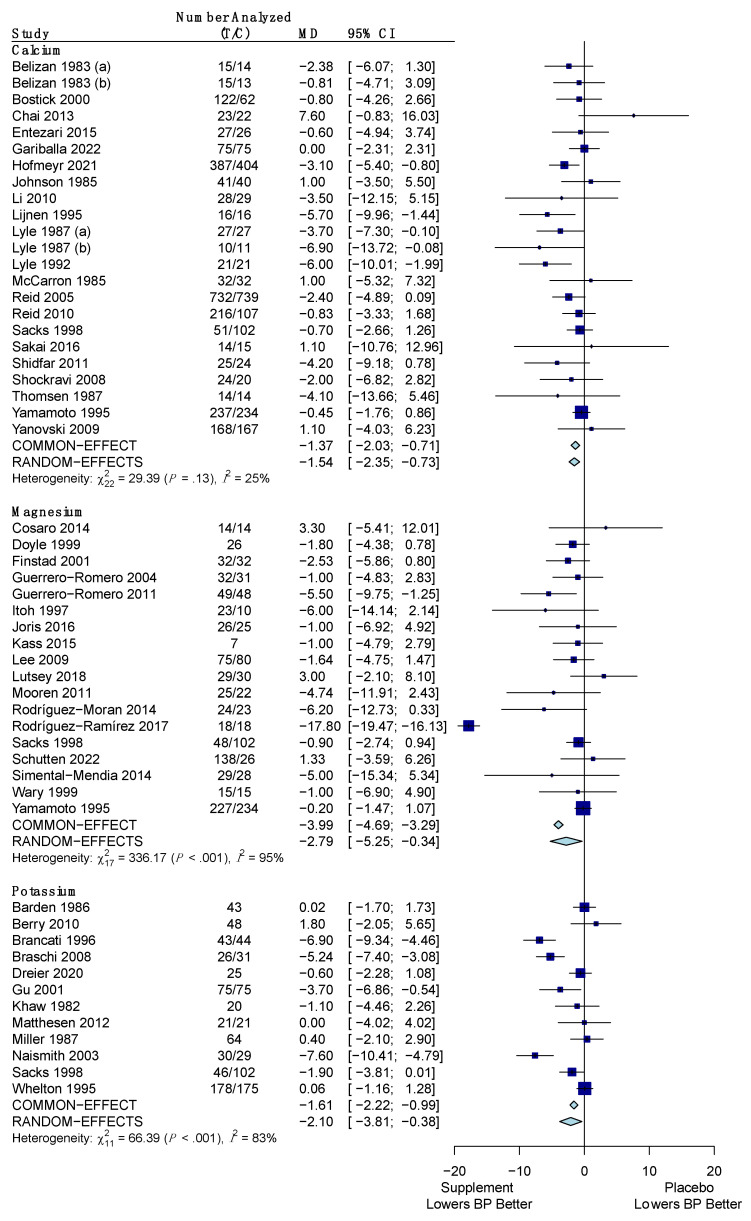
Forest plot showing the mean difference of change in systolic blood pressure between minerals (calcium, magnesium, potassium) and placebo. T represents the sample size of the treatment (supplement/mineral) group and C represents the sample size of the control (placebo) group. The pooled mean difference is given in mm Hg with its 95% CI. The weighted mean difference in mm Hg and its 95% CI is given for each study. A graphical representation with the weighted mean difference and its SE is also given for each study. References: Calcium ([37,39,43,52,55,66,67,70,71,73,91,92,95,96,101,102,106,114,116,119,123]); Magnesium ([45,46,48,54,58,59,60,65,69,76,93,94,95,98,103,109,114,118]); Potassium ([36,38,40,41,47,53,62,72,82,95,110,121]).

The pooled results for the difference in the change of SBP for the minerals versus placebo are shown in Figure 4. All three minerals (calcium, magnesium, and potassium) showed a significant reduction of SBP versus placebo. Calcium reduced SBP by an average of −1.37 mm Hg (95% CI: −2.03, −0.71) using a common effects model due to low heterogeneity (*I*^2^ = 25%; *p*-value: 0.13). Magnesium had an average reduction in SBP of −2.79 mm Hg (95% CI: −5.25, −0.34) using the random effects model due to high heterogeneity (*I*^2^ = 95%; *p*-value: < 0.001). Potassium also used the random effects model due to high heterogeneity (*I*^2^ = 83%; *p*-value: < 0.001) and was found to reduce SBP by an average of −2.10 mm Hg (95% CI: −3.81, −0.38).

#### 3.4.2. Diastolic Blood Pressure

**Figure 5:**
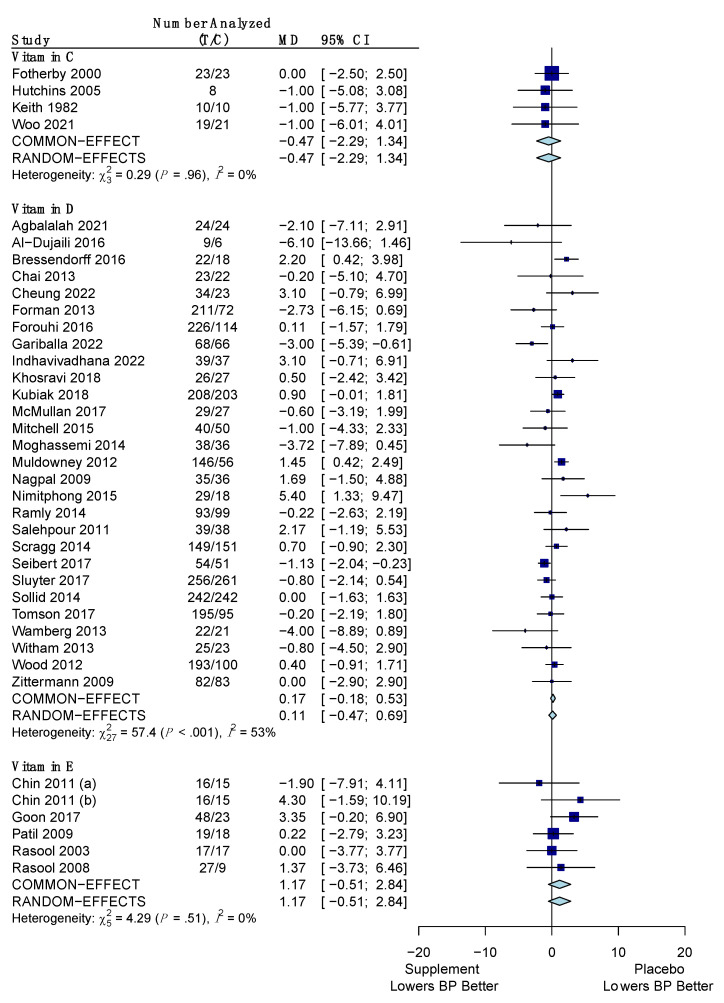
Forest plot showing the mean difference of change in diastolic blood pressure between vitamins (C, D, E) and placebo. T represents the sample size of the treatment (supplement/vitamin) group and C represents the sample size of the control (placebo) group. The pooled mean difference is given in mm Hg with its 95% CI. The weighted mean difference in mm Hg and its 95% CI is given for each study. A graphical representation with the weighted mean difference and its SE is also given for each study. References: Vitamin C ([51,56,61,112]); Vitamin D ([33,34,42,43,44,49,50,52,57,63,64,74,75,79,81,83,87,97,99,100,104,105,107,108,111,113,122,124]); Vitamin E ([86,88,90,115,117]).

The pooled results for the difference in the change of DBP for the vitamins versus placebo are shown in Figure 5. None of the three vitamins (C, D, E) were able to reduce DBP versus placebo. Vitamin C and E were deemed to have low heterogeneity (*I*^2^ = 0%; *p*-value: 0.96 and *I*^2^ = 0%; *p*-value: 0.51, respectively) and thus their effects on DBP were determined using the common effects model. These effects were −0.47 mm Hg (95% CI: −2.29, 1.34) and +1.17 mm Hg (95% CI: −0.51, 2.84), respectively. Vitamin D resulted in an increase in DBP versus placebo of +0.11 mm Hg (95% CI: −0.47, 0.69) using the random effects model due to high heterogeneity (*I*^2^ = 53%; *p*-value: < 0.001).

**Figure 6:**
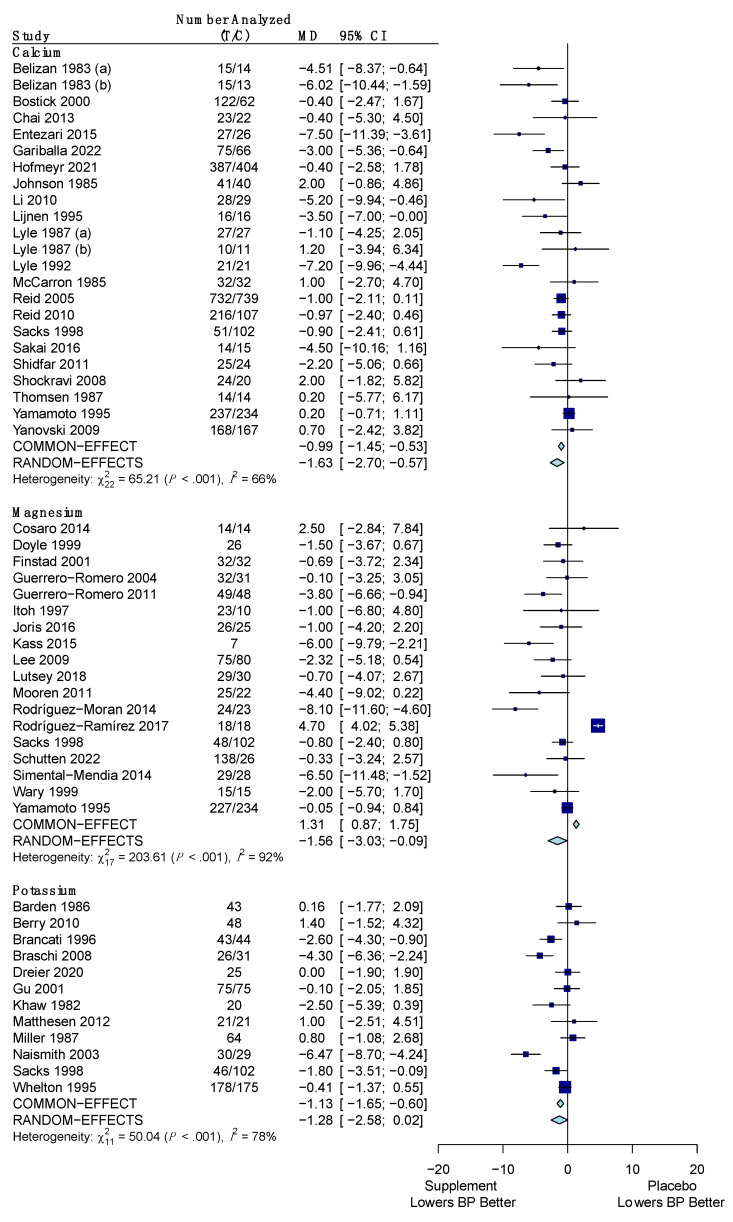
Forest plot showing the mean difference of change in diastolic blood pressure between minerals (calcium, magnesium, potassium) and placebo. T represents the sample size of the treatment (supplement/mineral) group and C represents the sample size of the control (placebo) group. The pooled mean difference is given in mm Hg with its 95% CI. The weighted mean difference in mm Hg and its 95% CI is given for each study. A graphical representation with the weighted mean difference and its SE is also given for each study. References: Calcium ([37,39,43,52,55,66,67,70,71,73,91,92,95,96,101,102,106,114,116,119,123]); Magnesium ([45,46,48,54,58,59,60,65,69,76,93,94,95,98,103,109,114,118]); Potassium ([36,38,40,41,47,53,62,72,82,95,110,121]).

The pooled results for the difference in the change of DBP for the minerals versus placebo are shown in Figure 6. These effects were calculated using the random effects model for all three minerals (calcium, magnesium, and potassium) due to high heterogeneity. Potassium was the only one of the three to have a statistically insignificant reduction in DBP, with a mean difference of −1.28 mm Hg (95% CI: −2.58, 0.02) and high heterogeneity (*I*^2^ = 78%; *p*-value: < 0.001). Calcium reduced DBP by a mean difference of −1.63 mm Hg (95% CI: −2.70, −0.57) with high heterogeneity amongst the studies (*I*^2^ = 66%; *p*-value: < 0.001). Magnesium had a mean difference in DBP of −1.56 mm Hg (95% CI: −3.03, −0.09), again with high heterogeneity (*I*^2^ = 92%; *p*-value: < 0.001).

#### 3.4.3. Summary

A summary of the effects of the vitamins and minerals versus placebo is provided in Table 3. All three minerals (calcium, magnesium, and potassium), as well as vitamin E, had a statistically significant mean reduction in SBP, whereas only calcium and magnesium had a statistically significant reduction in DBP. However, potassium was close to having a statistically significant mean reduction in DBP with the upper limit of its 95% CI being just above 0.

#### 3.4.4. Sensitivity Analysis

A sensitivity analysis was performed by imputing the missing standard deviations of change-from-baseline scores for parallel comparisons assuming a correlation coefficient of 0.5. For cross-over studies, the standard errors of effect sizes were calculated assuming correlation coefficients of 0.5 and 0.9. The results are summarized in Table S6. Overall, only the effect of magnesium on DBP reduction became nonsignificant when a smaller correlation coefficient was used to impute standard deviations of change-from-baseline scores.

#### 3.4.5. Publication Bias

Contour-enhanced funnel plots of the six supplements with their effect sizes on the horizontal axis and SEs on the vertical axis were constructed to visualize potential publication bias. These plots can be found by supplement for systolic and diastolic BP in Figures S1 and S2, respectively. The asymmetric patterns seen in the SBP analysis for vitamin E and the DBP analyses for calcium and magnesium indicate potential publication bias associated with these analyses. This was confirmed by Egger’s regression, yielding significant results for these three analyses (*p*-values of 0.075, 0.069, and 0.001, respectively). The DBP analysis for vitamin C was also noted to have publication bias with Egger’s regression (*p*-value: 0.036).

## 4. Discussion

Our results provide evidence that vitamin E, calcium, magnesium, and potassium are effective in lowering SBP in the general, normotensive population. Both magnesium and potassium achieved a greater than −2 mm Hg reduction in SBP, with reductions of −2.79 mm Hg and −2.10 mm Hg, respectively. These reductions can be considered clinically significant, as prior studies have found that a −2 mm Hg reduction in SBP could reduce mortality from heart disease and stroke by as much as 7% and 10%, respectively [4,125]. Our results also show that both calcium and magnesium are effective in lowering DBP in this population. Furthermore, although the DBP reduction of −1.28 mm Hg by potassium was not significant, the upper limit of its confidence interval was just above zero at 0.02. Overall, this study and its findings are important as no previous study has conducted meta-analyses on the effectiveness of multiple nutraceuticals in reducing BP amongst this population. We hope that our significant findings and accessibility of data will be of help to those interested in the BP-lowering capabilities of these nutraceuticals.

Our reductions in blood pressure were generally lower than the values in previously published meta-analyses of these nutraceuticals. This supports our hypothesis that these studies, which included subjects with uncontrolled hypertension who were receiving these nutraceuticals as an initial treatment, likely saw greater reductions in BP as a result. This discrepancy was largest for vitamin C, vitamin E, and potassium. For example, the most recent published meta-analysis on vitamin C yielded statistically significant reductions of both systolic and diastolic blood pressure of −4.09 mm Hg and −2.30 mm Hg [15]. This is a drastic contrast to our values of −1.45 mm Hg and −0.47 mm Hg, with neither being significant. While vitamin E provided a statistically significant reduction in SBP of −1.76 mm Hg in our study, this was lower than a 2019 meta-analysis of 18 RCTs that achieved a value of −3.4 mm Hg [17]. We also obtained an increase in DBP of 1.17 mm Hg compared to their reduction of −1.19 mm Hg, although neither result was deemed significant [17]. Similar differences existed with potassium. The most recent publication on potassium obtained reductions of −3.9 mm Hg and −2.4 mm Hg [20], nearly twice our values of −2.10 mm Hg and −1.28 mm Hg, the latter of which did not reach significance, as mentioned earlier. However, it is worth noting that we do not know whether the differences between our values and the most recent published meta-analyses are statistically significant since no analysis was performed to assess.

Our results for vitamin D and calcium were similar to those found in the most recent publications [16,18]. Upon further examination of these studies, we found that they targeted a similar normotensive population, which could explain the similarity in our findings. However, even outside of this controlled population, vitamin D has not shown to be effective at lowering systolic or diastolic BP [126,127]. This holds true even in individuals with vitamin D deficiency [128]. On the other hand, calcium has been consistently shown to reduce BP. Another meta-analysis of 40 trials with an average daily calcium dose of 1200 mg showed similar reductions to what we obtained in regard to SBP and DBP [129]. Although, they further noted that these reductions were more pronounced in people with lower baseline calcium intake [129]. Interestingly, a meta-analysis of 8 trials with vitamin D and calcium co-supplementation showed no significant reduction in SBP and only −0.23 mm Hg in DBP [130]. Further studies with larger sample sizes should be conducted before passing judgement on the efficacy of this combination.

Aside from calcium, magnesium was the only other nutraceutical to produce a significant reduction in both SBP and DBP. Notably, the −2.79 mm Hg reduction in SBP was greater than the −2.00 mm Hg seen in a meta-analysis of 34 trials that did not control for our general, normotensive population [19]. However, another study obtained vastly different results, concluding that magnesium supplementation does not lower BP in normotensives or controlled hypertensives, even at high doses [131]. Due to this discrepancy in the literature, future research is warranted, especially given the promising results that our study obtained. Aside from having larger sample sizes to ensure power, these studies should investigate a variety of dosages, given the large ranges noted in the trials we included. Additionally, longer treatment periods should be utilized, as the longest trial in our study was just six months.

Potassium was the only other nutraceutical to produce a reduction in SBP of at least −2 mm Hg. As noted, however, this reduction was only half of what has been seen in studies that were not controlling for a general, normotensive population [20]. This previous study also performed a dose-response meta-analysis that yielded a U-shaped graph, suggesting that while potassium can lower BP, higher dosages might increase it [20]. It is important to note that not only did the normotensive group experience smaller reductions in BP but they were also more sensitive to higher dosages of potassium, experiencing an increased BP at a dosage of 60 mmol/day versus 90 mmol/day in the hypertensive group [20]. Another study examining the effect of potassium on primary essential hypertension obtained similarly sized reductions in SBP and DBP to the previously mentioned one, but showed that higher dosages of potassium, specifically ≥100 mmol/day, achieved the greatest reductions [132]. Given the conflicting results between these studies, future studies should focus on the optimal dosage for various populations, as well as the long-term safety of potassium supplementation.

Although vitamin C and vitamin E yielded uninspiring results for BP reduction in this population, our study may have been limited by low power due to the relatively low number of eligible trials and their small sample sizes. Future studies with larger power should evaluate the efficacy of these two vitamins amongst this general, normotensive population, especially with their promising results in previous studies. If conducted, these studies should focus on vitamin C at a dosage of ≥500 mg/day and a duration of ≥6 weeks, as subgroup analysis in the prior meta-analysis found these to yield the greatest reductions in BP [15]. Vitamin E was found to achieve the greatest reduction at dosages ≤ 400 mg/day and their results were independent of treatment duration [17]. This data on vitamin E may be of interest since we did obtain a significant reduction in SBP in our study.

While we did not investigate ideal dosage and treatment length in our study, prior studies provide insight into these. A systematic review provided the recommended daily intake for the supplements used in our study [133]. These are 70–90 mg, 10–20 μg (400–800 IU), and 10–15 mg for vitamins C, D, and E, respectively [133]. Calcium, magnesium, and potassium have recommended daily values of 1000–1300 mg, 350–420 mg, and 4700–4800 mg, respectively [133]. The published meta-analyses with subgroup analyses can be used to further speculate on optimal dosage and treatment length in our population for those nutraceuticals that showed efficacy in our study. Calcium, which had a significant reduction in both SBP and DBP in our study, showed greatest efficacy at dosages > 1500 mg/day and treatment durations < 6 months [18]. Magnesium achieved greatest reductions in BP at dosages < 300 mg/day and with a duration of 30–89 days (1–3 months) [19]. The effects of potassium on BP have been shown to be independent of treatment duration, but the optimal dosage is still to be determined, as we mentioned above [20,132]. We intend to investigate ideal dosage and treatment length for this population in future studies to build upon our findings. Further subgroup analyses will also be considered at that time, such as into variations by age, gender, ethnicity, and past medical history to further refine recommendations into the use of these nutraceuticals. However, for the purposes of this study, we felt in-depth subgroup analyses across all six nutraceuticals in a single paper would complicate the reporting of our results and take away from the clear overview that we sought to provide.

Our results are important because they shed light onto what the general, normotensive population can take to achieve optimal BP. We are the first study to provide data on multiple vitamins and minerals for this purpose, allowing access for easy comparison of efficacy versus placebo in this population. We achieved high power with large sample sizes for vitamin D, calcium, magnesium, and potassium that we hope adds credibility to our results and promise for its generalizability. Furthermore, while proving to be effective in lowering BP, these six supplements are considered natural and part of a balanced diet. This may help appeal to individuals who are hesitant to take pharmaceuticals for controlling BP, although these supplements should not be viewed as alternatives in those requiring antihypertensives. Current clinical practice guidelines recommend initiating antihypertensives when SBP is ≥140 and/or DBP is ≥90 in patients with primary hypertension without other co-morbidities warranting sooner intervention [134,135]. Therefore, we hope our results aid in the decision-making for those who have BPs outside of this range but still above optimal. Specifically, our data should be used for individuals with BPs above the optimal range of 110–115/70–75 mm Hg that don’t qualify for initiation of prescription antihypertensives based on clinical practice guidelines. The goal of initiation of these vitamins and minerals would be to lower BP closer to this level, potentially reducing the increased mortality seen from vascular events and coronary heart disease at higher BPs in the process.

Additionally, no adverse events were reported in any of our 87 included trials, while mild side effects were seen with the supplement group in only six, with magnesium and vitamin D accounting for three each. These side effects included mild diarrhea in the three magnesium trials and a range of symptoms in the vitamin D trials from headache to abdominal pain to constipation/diarrhea. Overall, these supplements appear to be an inexpensive and safe option for better BP control when patients don’t qualify for antihypertensives. While all six would be eligible options for the general, normotensive population, particular attention should be given to the four (vitamin E, calcium, magnesium, and potassium) that showed efficacy in lowering BP. This is especially true for magnesium and potassium because of their clinically significant reduction in SBP, with magnesium exhibiting efficacy in lowering DBP also. Due to this efficacy, magnesium appears to be the most promising of our six supplements in this population.

Our study is not without limitations, the most glaring of which is that we did not control for subgroups, including differences in patient populations, dosages of supplements, and treatment length. While we had strict inclusion and exclusion criteria, differences in efficacy likely exist amongst patient population subgroups, as well as the various dosages and/or treatment lengths. Ultimately, we were willing to accept this limitation going into the study, as we hoped this paper could serve mainly as a preliminary, foundational analysis of how these six nutraceuticals could be used amongst a general, normotensive population. The interpretation of our results is thereby limited, as we can only state the mean reductions in SBP and DBP for the supplements against placebo across a range of patient populations, dosages, and treatment lengths. However, we hope that our compiled references, data points, and findings will be used to guide more specific analyses in the future. As mentioned, future studies should investigate optimal dosage and treatment length, as well as the long-term efficacy and safety of these supplements. Priority should be given to those that showed significant reductions in this population: vitamin E, calcium, magnesium, and potassium. Combinations of supplements could also be explored to explore for additive and/or synergistic effects, as we excluded these studies in our study to determine the individual efficacy of each nutraceutical versus placebo.

Additionally, publication bias was noted in some of our analyses, as evidenced by the asymmetry in their funnel plots. This occurred in the meta-analyses of SBP with vitamin E and DBP for calcium and magnesium, with confirmation for all three provided by Egger’s regression. The Egger’s regression also highlighted potential publication bias in the meta-analysis of DBP for vitamin C. Given these risks of bias, the pooled results of these analyses should be considered with caution. On the same token, significant heterogeneity was seen in certain analyses, thus its effect on the analyses cannot be ignored when considering the validity of the results.

## 5. Conclusions

In conclusion, to our knowledge, this is the first study to run pairwise meta-analyses on the BP lowering capacity of all six of these nutraceuticals versus placebo in the general, normotensive population. Our results displayed evidence for vitamin E, calcium, magnesium, and potassium being effective at lowering SBP in this population, with both magnesium and potassium achieving a greater than 2 mm Hg reduction. Additionally, calcium and magnesium were determined to be effective at lowering DBP in this population. Given these findings, magnesium seems to be the most effective of the six nutraceuticals studied in lowering BP in this population. Future studies should look further into the use of these nutraceuticals to determine optimal dosage and treatment length, long-term safety and efficacy, and potential additive and/or synergistic effects.

## Figures and Tables

**Table 1:** Summary of the basic characteristics of included studies broken down by supplement.

Supplement (Total Number of Studies)	Type of Studies	Data	Sample Size	Population	Dose Range	Treatment Length Range
Vitamin C (*n* = 5)	P: 1 (20%) CO: 4 (80%)	Sys: 5 (100%) Dia: 4 (80%)	Sys: 122 Dia: 114	Healthy: 4 (80%) General: 1 (20%)	200–1000 mg/day	2–52 weeks
Vitamin D (*n* = 29)	P: 29 (100%) CO: 0 (0%)	Sys: 29 (100%) Dia: 28 (96.6%)	Sys: 4897 Dia: 4578	Healthy: 9 (31.0%) General: 8 (27.6%) Obese: 8 (27.6%) Postmenopausal: 4 (13.8%)	200–8000 IU/day	2–208 weeks
Vitamin E (*n* = 7)	P: 5 (71.4%) CO: 2 (28.6%)	Sys: 7 (100%) Dia: 5 (71.4%)	Sys: 302 Dia: 240	Healthy: 6 (85.7%) Postmenopausal: 1 (14.3%)	50–320 mg/day	3–26 weeks
Calcium (*n* = 21)	P: 20 (95.2%) CO: 1 (4.8%)	Sys: 21 (100%) Dia: 21 (100%)	Sys: 4534 Dia: 4525	Healthy: 12 (57.1%) General: 3 (14.3%) Obese: 3 (14.3%) Postmenopausal: 3 (14.3%)	162–2000 mg/day	4–208 weeks
Magnesium (*n* = 18)	P: 14 (77.8%) CO: 4 (22.2%)	Sys: 18 (100%) Dia: 18 (100%)	Sys: 1575 Dia: 1575	Healthy: 11 (61.1%) Obese: 4 (22.2%) General: 3 (16.7%)	212–636 mg/day	4–26 weeks
Potassium (*n* = 12)	P: 7 (58.3%) CO: 5 (41.7%)	Sys: 12 (100%) Dia: 12 (100%)	Sys: 1096 Dia: 1096	Healthy: 7 (58.3%) General: 5 (41.7%)	24–100 mmol/day (938.4–3910 mg/day)	3–26 weeks
Total (*n* = 87)	P: 71 (81.6%) CO: 16 (18.4%)	Sys: 87 (100%) Dia: 83 (95.4%)	Sys: 12,526 Dia: 12,128	Healthy: 44 (50.6%) General: 20 (23.0%) Obese: 15 (17.2%) Postmenopausal: 8 (9.2%)		

The number of parallel versus cross-over studies, number of studies reporting systolic and diastolic data, and number of each type of population is given in *n* (%), representing the frequency of findings. The denominator for this frequency is noted in the first column. Dosage and treatment length are given as ranges. P represents parallel trials, CO represents cross-over trials, Sys represents systolic blood pressure data, and Dia represents diastolic blood pressure data.

**Table 2:** Overall risk of bias by supplement. N represents the total number of studies for each supplement.

Supplement	Overall Risk of Bias
Vitamin C (*n* = 5)	Low: 4 (80%) Some concerns: 1 (20%) High: 0 (0%)
Vitamin D (*n* = 29)	Low: 25 (86.2%) Some concerns: 3 (10.3%) High: 1 (3.4%)
Vitamin E (*n* = 7)	Low: 6 (85.7%) Some concerns: 0 (0%) High: 1 (14.3%)
Calcium (*n* = 21)	Low: 17 (81.0%) Some concerns: 2 (9.5%) High: 2 (9.5%)
Magnesium (*n* = 18)	Low: 17 (94.4%) Some concerns: 1 (5.6%) High: 0 (0%)
Potassium (*n* = 12)	Low: 9 (75%) Some concerns: 2 (16.7%) High: 1 (8.3%)

The number of studies obtaining each of the three grades is noted in Column 2, with the percentages reflecting the frequency of each assignment for each supplement.

**Table 3:** Summary of the effects of vitamins (C, D, E) and minerals (calcium, magnesium, potassium) versus placebo on systolic and diastolic blood pressure.

Supplement	Systolic	Diastolic
Vitamin C	−1.45 mm Hg (−4.26, 1.35)	−0.47 mm Hg (−2.29, 1.34)
Vitamin D	−0.19 mm Hg (−0.71, 0.33)	+0.11 mm Hg (−0.47, 0.69)
Vitamin E	−1.76 mm Hg (−3.05, −0.47) *	+1.17 mm Hg (−0.51, 2.84)
Calcium	−1.37 mm Hg (−2.03, −0.71) *	−1.63 mm Hg (−2.70, −0.57) *
Magnesium	−2.79 mm Hg (−5.25, −0.34) *	−1.56 mm Hg (−3.03, −0.09) *
Potassium	−2.10 mm Hg (−3.81, −0.38) *	−1.28 mm Hg (−2.58, 0.02)

Values are mean systolic and diastolic blood pressure reductions for each supplement versus placebo with their corresponding 95% Confidence Intervals. Asterisks (*) denote statistical significance.

## Data Availability

The data presented in this study is available upon request from the corresponding author.

## Supplementary Materials

The following supporting information can be downloaded at: https://www.mdpi.com/article/10.3390/nu15194223/s1, Figure S1: Contour-enhanced funnel plots of the six meta-analyses in systolic blood pressure reduction; Figure S2: Contour-enhanced funnel plots of the six meta-analyses in diastolic blood pressure reduction; Table S1: Search terms for each of the four databases; Table S2: Reasons for exclusion of full-text articles (*n* = 325); Table S3: Reasons for exclusion of the five studies that met eligibility; Table S4: Basic characteristics of included studies; Table S5: Risk of bias results by individual study; Table S6: Results of sensitivity analyses for each supplement.

## References

[1] Mills K.T., Stefanescu A., He J. (2020). The Global Epidemiology of Hypertension. Nat. Rev. Nephrol..

[2] Kochanek K.D., Murphy S.L., Xu J., Arias E. (2019). Deaths: Final Data for 2017 pdf Icon [PDF—1.76 MB]. National Vital Statistics Reports.

[3] Whelton P.K., Carey R.M., Aronow W.S., Casey D.E., Collins K.J., Dennison Himmelfarb C., DePalma S.M., Gidding S., Jamerson K.A., Jones D.W. (2018). 2017 ACC/AHA/AAPA/ABC/ACPM/AGS/APhA/ASH/ASPC/NMA/PCNA Guideline For The Prevention, Detection, Evaluation, And Management of High Blood Pressure in Adults: Executive Summary: A Report of The American College of Cardiology/American Heart Association Task Force on Clinical Practice Guidelines. J. Am. Coll. Cardiol..

[4] Lissner L. (2002). Prospective Studies Collaboration. Age-Specific Relevance of Usual Blood Pressure to Vascular Mortality: A Meta-Analysis of Individual Data For One Million Adults in 61 Prospective Studies. Lancet (Br. Ed.).

[5] Law M.R., Morris J.K., Wald N.J. (2009). Use of Blood Pressure Lowering Drugs in the Prevention of Cardiovascular Disease: Meta-Analysis of 147 Randomised Trials in the Context of Expectations From Prospective Epidemiological Studies. BMJ.

[6] Borghi C., Cicero A.F.G. (2017). Nutraceuticals with A Clinically Detectable Blood Pressure-Lowering Effect: A Review of Available Randomized Clinical Trials and Their Meta-Analyses. Br. J. Clin. Pharmacol..

[7] Chiu H.F., Venkatakrishnan K., Golovinskaia O., Wang C.K. (2021). Impact of Micronutrients on Hypertension: Evidence from Clinical Trials with A Special Focus on Meta-Analysis. Nutrients.

[8] Huang A., Vita J.A., Venema R.C., Keaney J.F. (2000). Ascorbic Acid Enhances Endothelial Nitric-Oxide Synthase Activity By Increasing Intracellular Tetrahydrobiopterin. J. Biol. Chem..

[9] Sen C.K., Rink C., Khanna S. (2010). Palm Oil-Derived Natural Vitamin E A-Tocotrienol In Brain Health And Disease. J. Am. Coll. Nutr..

[10] Raghuvanshi R., Chandra M., Mishra A., Misra M.K. (2007). Effect of Vitamin E Administration on Blood Pressure Following Reperfusion of Patients With Myocardial Infarction. Exp. Clin. Cardiol..

[11] Forman J.P., Williams J.S., Fisher N.D.L. (2010). Plasma 25-Hydroxyvitamin D and Regulation of the Renin-Angiotensin System in Humans. Hypertension.

[12] Villa-Etchegoyen C., Lombarte M., Matamoros N., Belizán J.M., Cormick G. (2019). Mechanisms Involved in the Relationship Between Low Calcium Intake and High Blood Pressure. Nutrients.

[13] Cunha A.R., Umbelino B., Correia M.L., Neves M.F. (2012). Magnesium and Vascular Changes in Hypertension. Int. J. Hypertens..

[14] Poulsen S.B., Fenton R.A. (2019). K+ and the Renin–angiotensin–aldosterone System: New Insights into Their Role in Blood Pressure Control and Hypertension Treatment. J. Physiol..

[15] Guan Y., Dai P., Wang H. (2020). Effects of Vitamin C Supplementation on Essential Hypertension: A Systematic Review and Meta-Analysis. Medicine.

[16] Zhang D., Cheng C., Wang Y., Sun H., Yu S., Xue Y., Liu Y., Li W., Li X. (2020). Effect of Vitamin D on Blood Pressure And Hypertension In The General Population: An Update Meta-Analysis of Cohort Studies And Randomized Controlled Trials. Prev. Chronic Dis..

[17] Emami M.R., Safabakhsh M., Alizadeh S., Asbaghi O., Khosroshahi M.Z. (2019). Effect of Vitamin E Supplementation on Blood Pressure: A Systematic Review and Meta-Analysis. J. Hum. Hypertens..

[18] Cormick G., Ciapponi A., Cafferata M.L., Cormick M.S., Belizán J.M. (2022). Calcium Supplementation for Prevention of Primary Hypertension. Cochrane Database Syst. Rev..

[19] Zhang X., Li Y., Del Gobbo L.C., Rosanoff A., Wang J., Zhang W., Song Y. (2016). Effects of Magnesium Supplementation on Blood Pressure: A Meta-Analysis of Randomized Double-Blind Placebo-Controlled Trials. Hypertension.

[20] Filippini T., Naska A., Kasdagli M.I., Torres D., Lopes C., Carvalho C., Moreira P., Malavolti M., Orsini N., Whelton P.K. (2020). Potassium Intake and Blood Pressure: A Dose-Response Meta-Analysis of Randomized Controlled Trials. J. Am. Heart Assoc..

[21] Page M.J., McKenzie J.E., Bossuyt P.M., Boutron I., Hoffmann T.C., Mulrow C.D., Shamseer L., Tetzlaff J.M., Akl E.A., Brennan S.E. (2021). The PRISMA 2020 Statement: An Updated Guideline for Reporting Systematic Reviews. BMJ.

[22] (2022). Covidence Systematic Review Software, Veritas Health Innovation, Melbourne, Australia [Internet]. www.covidence.org.

[23] Higgins J.P.T., Savović J., Page M.J., Elbers R.G., Sterne J.A.C. (2019). Assessing Risk of Bias in a Randomized Trial. Cochrane Handbook for Systematic Reviews of Interventions.

[24] Higgins J.P.T., Li T., Deeks J.J. (2019). Choosing Effect Measures and Computing Estimates of Effect. Cochrane Handbook For Systematic Reviews of Interventions.

[25] Higgins J.P.T., Eldridge S., Li T. (2019). Including Variants on Randomized Trials. Cochrane Handbook For Systematic Reviews of Interventions.

[26] Cochran W.G. (1954). The Combination of Estimates From Different Experiments. Biometrics.

[27] Higgins J.P.T., Thompson S.G., Deeks J.J., Altman D.G. (2003). Measuring Inconsistency in Meta-Analyses. BMJ.

[28] Langan D., Higgins J.P.T., Jackson D., Bowden J., Veroniki A.A., Kontopantelis E., Viechtbauer W., Simmonds M. (2019). A Comparison of Heterogeneity Variance Estimators in Simulated Random-Effects Meta-Analyses. Res. Synth. Methods.

[29] Song F., Gilbody S. (1998). Bias In Meta-Analysis Detected By A Simple, Graphical Test. Increase in Studies of Publication Bias Coincided with Increasing Use of Meta-Analysis. BMJ.

[30] Light R.J., Pillemer D.B. (1984). Summing Up: The Science of Reviewing Research.

[31] Meng Z., Wu C., Lin L. (2023). The Effect Direction Should Be Taken into Account When Assessing Small-Study Effects. J. Evid. -Based Dent. Pract..

[32] Schwarzer G., Carpenter J.R., Rücker G. (2015). Meta-Analysis With R.

[33] Agbalalah T., Mushtaq S. (2022). Effect of vitamin D3 supplementation on cardiometabolic disease risk among overweight/obese adult males in United Kingdom-A pilot randomised controlled trial. J. Hum. Nutr. Diet..

[34] Al-Dujaili E.A.S., Munir N., Iniesta R.R. (2016). Effect of vitamin D supplementation on cardiovascular disease risk factors and exercise performance in healthy participants: A randomized placebo-controlled preliminary study. Ther. Adv. Endocrinol. Metab..

[35] Aringazina R., Kurmanalina G., Kurmanalin B., Degtyarevskaya T. (2021). Role of vitamin d in prevention of metabolic syndrome and cardiovascular diseases. Bangladesh J. Med. Sci..

[36] Barden A.E., Vandongen R., Beilin L.J., Margetts B., Rogers P. (1986). Potassium supplementation does not lower blood pressure in normotensive women. J. Hypertens..

[37] Belizan J.M., Villar J., Pineda O., Gonzalez A.E., Sainz E., Garrera G., Sibrian R. (1983). Reduction of blood pressure with calcium supplementation in young adults. JAMA.

[38] Berry S.E., Mulla U.Z., Chowienczyk P.J., Sanders T.A. (2010). Increased potassium intake from fruit and vegetables or supplements does not lower blood pressure or improve vascular function in UK men and women with early hypertension: A randomised controlled trial. Br. J. Nutr..

[39] Bostick R.M. (2000). Effect of calcium supplementation on serum cholesterol and blood pressure: A randomized, double-blind, placebo-controlled, clinical trial. Arch. Fam. Med..

[40] Brancati F.L., Appel L.J., Seidler A.J., Whelton P.K. (1996). Effect of potassium supplementation on blood pressure in African Americans on a low-potassium diet. A randomized, double-blind, placebo-controlled trial. Arch. Intern. Med..

[41] Braschi A., Naismith D.J. (2008). The effect of a dietary supplement of potassium chloride or potassium citrate on blood pressure in predominantly normotensive volunteers. Br. J. Nutr..

[42] Bressendorff I., Brandi L., Schou M., Nygaard B., Frandsen N.E., Rasmussen K., Ødum L., Østergaard O.V., Hansen D. (2016). The Effect of High Dose Cholecalciferol on Arterial Stiffness and Peripheral and Central Blood Pressure in Healthy Humans: A Randomized Controlled Trial. PLoS ONE.

[43] Chai W., Cooney R.V., Franke A.A., Bostick R.M. (2013). Effects of calcium and vitamin D supplementation on blood pressure and serum lipids and carotenoids: A randomized, double-blind, placebo-controlled, clinical trial. Ann. Epidemiol..

[44] Cheung M.M., Dall R.D., Shewokis P.A., Altasan A., Volpe S.L., Amori R., Singh H., Sukumar D. (2022). The effect of combined magnesium and vitamin D supplementation on vitamin D status, systemic inflammation, and blood pressure: A randomized double-blinded controlled trial. Nutrition.

[45] Cosaro E., Bonafini S., Montagnana M., Danese E., Trettene M.S., Minuz P., Delva P., Fava C. (2014). Effects of magnesium supplements on blood pressure, endothelial function and metabolic parameters in healthy young men with a family history of metabolic syndrome. Nutr. Metab. Cardiovasc. Dis..

[46] Doyle L., Flynn A., Cashman K. (1999). The effect of magnesium supplementation on biochemical markers of bone metabolism or blood pressure in healthy young adult females. Eur. J. Clin. Nutr..

[47] Dreier R., Abdolalizadeh B., Asferg C.L., Hölmich L.R., Buus N.H., Forman J.L., Andersen U.B., Egfjord M., Sheykhzade M., Jeppesen J.L. (2020). Effect of increased potassium intake on the renin-angiotensin-aldosterone system and subcutaneous resistance arteries: A randomized crossover study. Nephrol. Dial. Transplant..

[48] Finstad E.W., Newhouse I.J., Lukaski H.C., McAuliffe J.E., Stewart C.R. (2001). The effects of magnesium supplementation on exercise performance. Med. Sci. Sports Exerc..

[49] Forman J.P., Scott J.B., Ng K., Drake B.F., Suarez E.G., Hayden D.L., Bennett G.G., Chandler P.D., Hollis B.W., Emmons K.M. (2013). Effect of vitamin D supplementation on blood pressure in blacks. Hypertension.

[50] Forouhi N.G., Menon R.K., Sharp S.J., Mannan N., Timms P.M., Martineau A.R., Rickard A.P., Boucher B.J., Chowdhury T.A., Griffiths C.J. (2016). Effects of vitamin D2 or D3 supplementation on glycaemic control and cardiometabolic risk among people at risk of type 2 diabetes: Results of a randomized double-blind placebo-controlled trial. Diabetes Obes. Metab..

[51] Fotherby M.D., Williams J.C., Forster L.A., Craner P., Ferns G.A. (2000). Effect of vitamin C on ambulatory blood pressure and plasma lipids in older persons. J. Hypertens..

[52] Gariballa S., Yasin J., Alessa A. (2022). A randomized, double-blind, placebo-controlled trial of vitamin D supplementation with or without calcium in community-dwelling vitamin D deficient subjects. BMC Musculoskelet. Disord..

[53] Gu D., He J., Wu X., Duan X., Whelton P.K. (2001). Effect of potassium supplementation on blood pressure in Chinese: A randomized, placebo-controlled trial. J. Hypertens..

[54] Guerrero-Romero F., Tamez-Perez H.E., González-González G., Salinas-Martínez A.M., Montes-Villarreal J., Treviño-Ortiz J.H., Rodríguez-Morán M. (2004). Oral magnesium supplementation improves insulin sensitivity in non-diabetic subjects with insulin resistance. A double-blind placebo-controlled randomized trial. Diabetes Metab..

[55] Hofmeyr G.J., Seuc A., Betrán A.P., Cormick G., Singata M., Fawcus S., Mose S., Frank K., Hall D., Belizán J. (2021). The effect of calcium supplementation on blood pressure in non-pregnant women with previous pre-eclampsia: A randomized placebo-controlled study. Pregnancy Hypertens. Int. J. Women’s Cardiovasc. Health.

[56] Hutchins A.M., McIver I.E., Johnston C.S. (2005). Soy isoflavone and ascorbic acid supplementation alone or in combination minimally affect plasma lipid peroxides in healthy postmenopausal women. J. Am. Diet. Assoc..

[57] Indhavivadhana S., Boonyachan W., Rattanachaiyanont M., Wongwananuruk T., Techatraisak K., Sa-nga-areekul N. (2022). Effectiveness of vitamin D2 supplementation on high-sensitivity C-reactive protein and other metabolic indices in menopausal Thai women: A randomized-controlled trial. Gynecol. Endocrinol..

[58] Itoh K., Kawasaka T., Nakamura M. (1997). The effects of high oral magnesium supplementation on blood pressure, serum lipids and related variables in apparently healthy Japanese subjects. Br. J. Nutr..

[59] Joris P.J., Plat J., Bakker S.J., Mensink R.P. (2016). Long-term magnesium supplementation improves arterial stiffness in overweight and obese adults: Results of a randomized, double-blind, placebo-controlled intervention trial. Am. J. Clin. Nutr..

[60] Kass L.S., Poeira F. (2015). The effect of acute vs chronic magnesium supplementation on exercise and recovery on resistance exercise, blood pressure and total peripheral resistance on normotensive adults. J. Int. Soc. Sports Nutr..

[61] Keith R.E., Driskell J.A. (1982). Lung function and treadmill performance of smoking and nonsmoking males receiving ascorbic acid supplements. Am. J. Clin. Nutr..

[62] Khaw K.T., Thom S. (1982). Randomised double-blind cross-over trial of potassium on blood-pressure in normal subjects. Lancet.

[63] Khosravi Z.S., Kafeshani M., Tavasoli P., Zadeh A.H., Entezari M.H. (2018). Effect of Vitamin D supplementation on weight loss, glycemic indices, and lipid profile in obese and overweight women: A clinical trial study. Int. J. Prev. Med..

[64] Kubiak J., Thorsby P.M., Kamycheva E., Jorde R. (2018). Vitamin D supplementation does not improve CVD risk factors in vitamin D-insufficient subjects. Endocr. Connect..

[65] Lee S., Park H.K., Son S.P., Lee C.W., Kim I.J., Kim H.J. (2009). Effects of oral magnesium supplementation on insulin sensitivity and blood pressure in normo-magnesemic nondiabetic overweight Korean adults. Nutr. Metab. Cardiovasc. Dis..

[66] Li Y., Wang C., Zhu K., Feng R.N., Sun C.H. (2010). Effects of multivitamin and mineral supplementation on adiposity, energy expenditure and lipid profiles in obese Chinese women. Int. J. Obes..

[67] Lijnen P., Petrov V. (1995). Dietary calcium, blood pressure and cell membrane cation transport systems in males. J. Hypertens..

[68] Luft F.C., Aronoff G.R., Fineberg N.S., Weinberger M.H. (1989). Effects of oral calcium, potassium, digoxin, and nifedipine on natriuresis in normal humans. Am. J. Hypertens..

[69] Lutsey P.L., Chen L.Y., Eaton A., Jaeb M., Rudser K.D., Neaton J.D., Alonso A. (2018). A Pilot Randomized Trial of Oral Magnesium Supplementation on Supraventricular Arrhythmias. Nutrients.

[70] Lyle R.M., Melby C.L., Hyner G.C., Edmondson J.W., Miller J.Z., Weinberger M.H. (1987). Blood pressure and metabolic effects of calcium supplementation in normotensive white and black men. JAMA.

[71] Lyle R.M. (1992). Does baseline serum total calcium level influence the blood pressure response to calcium supplementation? A double-blind study. Neth. J. Med..

[72] Matthesen S.K., Larsen T., Vase H., Lauridsen T.G., Pedersen E.B. (2012). Effect of potassium supplementation on renal tubular function, ambulatory blood pressure and pulse wave velocity in healthy humans. Scand. J. Clin. Lab. Investig..

[73] McCarron D.A., Morris C.D. (1985). Blood pressure response to oral calcium in persons with mild to moderate hypertension. A randomized, double-blind, placebo-controlled, crossover trial. Annu. Intern. Med..

[74] McMullan C.J., Borgi L., Curhan G.C., Fisher N., Forman J.P. (2017). The effect of vitamin D on renin-angiotensin system activation and blood pressure: A randomized control trial. J. Hypertens..

[75] Mitchell D.M., Leder B.Z., Cagliero E., Mendoza N., Henao M.P., Hayden D.L., Finkelstein J.S., Burnett-Bowie S.A. (2015). Insulin secretion and sensitivity in healthy adults with low vitamin D are not affected by high-dose ergocalciferol administration: A randomized controlled trial. Am. J. Clin. Nutr..

[76] Mooren F.C., Krüger K., Völker K., Golf S.W., Wadepuhl M., Kraus A. (2011). Oral magnesium supplementation reduces insulin resistance in non-diabetic subjects—A double-blind, placebo-controlled, randomized trial. Diabetes Obes. Metab..

[77] el-D Mostafa S., Garner D.D., Garrett L., Whaley R.F., el-Sekate M., Kiker M. (1989). Beneficial effects of vitamin C on risk factors of cardiovascular diseases. J. Egypt. Public Health Assoc..

[78] Mottram P., Shige H., Nestel P. (1999). Vitamin E improves arterial compliance in middle-aged men and women. Atherosclerosis.

[79] Muldowney S., Lucey A.J., Hill T.R., Seamans K.M., Taylor N., Wallace J.M., Horigan G., Barnes M.S., Bonham M.P., Duffy E.M. (2012). Incremental cholecalciferol supplementation up to 15 μg/d throughout winter at 51-55° N has no effect on biomarkers of cardiovascular risk in healthy young and older adults. J. Nutr..

[80] Mullen J.T., O’Connor D.T. (1990). Potassium effects on blood pressure: Is the conjugate anion important?. J. Hum. Hypertens..

[81] Nagpal J., Pande J.N., Bhartia A. (2009). A double-blind, randomized, placebo-controlled trial of the short-term effect of vitamin D3 supplementation on insulin sensitivity in apparently healthy, middle-aged, centrally obese men. Diabet. Med..

[82] Naismith D.J., Braschi A. (2003). The effect of low-dose potassium supplementation on blood pressure in apparently healthy volunteers. Br. J. Nutr..

[83] Nimitphong H., Samittarucksa R., Saetung S., Bhirommuang N., Chailurkit L.O., Ongphiphadhanakul B. (2015). The Effect of Vitamin D Supplementation on Metabolic Phenotypes in Thais with Prediabetes. J. Med. Assoc. Thail..

[84] Nowson C., Morgan T. (1989). Effect of calcium carbonate on blood pressure in normotensive and hypertensive people. Hypertension.

[85] Osilesi O., Trout D.L., Ogunwole J.O., Glover E.E. (1991). Blood pressure and plasma lipids during ascorbic acid supplementation in borderline hypertensive and normotensive adults. Nutr. Res..

[86] Patil S.M., Chaudhuri D., Dhanakshirur G.B. (2009). Role of alpha-tocopherol in cardiopulmonary fitness in endurance athletes, cyclists. Indian J. Physiol. Pharmacol..

[87] Ramly M., Ming M.F., Chinna K., Suboh S., Pendek R. (2014). Effect of vitamin D supplementation on cardiometabolic risks and health-related quality of life among urban premenopausal women in a tropical country--a randomized controlled trial. PLoS ONE.

[88] Rasool A.H., Rehman A., Wan Yusuf W.N., Rahman A.R. (2003). Vitamin E and its effect on arterial stiffness in postmenopausal women--a randomized controlled trial. Int. J. Clin. Pharmacol. Ther..

[89] Rasool A.H., Yuen K.H., Yusoff K., Wong A.R., Rahman A.R. (2006). Dose dependent elevation of plasma tocotrienol levels and its effect on arterial compliance, plasma total antioxidant status, and lipid profile in healthy humans supplemented with tocotrienol rich vitamin E. J. Nutr. Sci. Vitaminol..

[90] Rasool A.H., Rahman A.R., Yuen K.H., Wong A.R. (2008). Arterial compliance and vitamin E blood levels with a self emulsifying preparation of tocotrienol rich vitamin E. Arch. Pharmacal Res..

[91] Reid I.R., Horne A., Mason B., Ames R., Bava U., Gamble G.D. (2005). Effects of calcium supplementation on body weight and blood pressure in normal older women: A randomized controlled trial. J. Clin. Endocrinol. Metab..

[92] Reid I.R., Ames R., Mason B., Bolland M.J., Bacon C.J., Reid H.E., Kyle C., Gamble G.D., Grey A., Horne A. (2010). Effects of calcium supplementation on lipids, blood pressure, and body composition in healthy older men: A randomized controlled trial. Am. J. Clin. Nutr..

[93] Rodríguez-Moran M., Guerrero-Romero F. (2014). Oral magnesium supplementation improves the metabolic profile of metabolically obese, normal-weight individuals: A randomized double-blind placebo-controlled trial. Arch. Med. Res..

[94] Rodríguez-Ramírez M., Rodríguez-Morán M., Reyes-Romero M.A., Guerrero-Romero F. (2017). Effect of oral magnesium supplementation on the transcription of TRPM6, TRPM7, and SLC41A1 in individuals newly diagnosed of pre-hypertension. A randomized, double-blind, placebo-controlled trial. Magnes. Res..

[95] Sacks F.M., Willett W.C., Smith A., Brown L.E., Rosner B., Moore T.J. (1998). Effect on blood pressure of potassium, calcium, and magnesium in women with low habitual intake. Hypertension.

[96] Sakai S., Hien V.T.T., Tuyen L.D., Duc H.A., Masuda Y., Yamamoto S. (2017). Effects of Eggshell Calcium Supplementation on Bone Mass in Postmenopausal Vietnamese Women. J. Nutr. Sci. Vitaminol..

[97] Salehpour A., Shidfar F., Hosseinpanah F., Vafa M., Razaghi M., Hoshiarrad A., Gohari M. (2012). Vitamin D3 and the risk of CVD in overweight and obese women: A randomised controlled trial. Br. J. Nutr..

[98] Schutten J.C., Joris P.J., Groendijk I., Eelderink C., Groothof D., van der Veen Y., Westerhuis R., Goorman F., Danel R.M., de Borst M.H. (2022). Effects of Magnesium Citrate, Magnesium Oxide, and Magnesium Sulfate Supplementation on Arterial Stiffness: A Randomized, Double-Blind, Placebo-Controlled Intervention Trial. J. Am. Heart Assoc..

[99] Scragg R., Slow S., Stewart A.W., Jennings L.C., Chambers S.T., Priest P.C., Florkowski C.M., Camargo C.A., Murdoch D.R. (2014). Long-term high-dose vitamin D3 supplementation and blood pressure in healthy adults: A randomized controlled trial. Hypertension.

[100] Seibert E., Lehmann U., Riedel A., Ulrich C., Hirche F., Brandsch C., Dierkes J., Girndt M., Stangl G.I. (2017). Vitamin D_3_ supplementation does not modify cardiovascular risk profile of adults with inadequate vitamin D status. Eur. J. Nutr..

[101] Shidfar F., Moghayedi M., Kerman S.R.J., Hosseini S., Shidfar S. (2011). Effects of a calcium supplement on serum lipoproteins, apolipoprotein B, and blood pressure in overweight men. Int. J. Endocrinol. Metab..

[102] Shockravi S., Jalali M.T., Rokn M., Karandish M. (2008). Effect of calcium supplementation on blood pressure in overweight or obese women. J. Biol. Sci..

[103] Simental-Mendía L.E., Rodríguez-Morán M., Guerrero-Romero F. (2014). Oral magnesium supplementation decreases C-reactive protein levels in subjects with prediabetes and hypomagnesemia: A clinical randomized double-blind placebo-controlled trial. Arch. Med. Res..

[104] Sluyter J.D., Camargo C.A., Stewart A.W., Waayer D., Lawes C.M.M., Toop L., Khaw K.T., Thom S.A.M., Hametner B., Wassertheurer S. (2017). Effect of Monthly, High-Dose, Long-Term Vitamin D Supplementation on Central Blood Pressure Parameters: A Randomized Controlled Trial Substudy. J. Am. Heart Assoc..

[105] Sollid S.T., Hutchinson M.Y., Fuskevåg O.M., Figenschau Y., Joakimsen R.M., Schirmer H., Njølstad I., Svartberg J., Kamycheva E., Jorde R. (2014). No effect of high-dose vitamin D supplementation on glycemic status or cardiovascular risk factors in subjects with prediabetes. Diabetes Care.

[106] Thomsen K., Nilas L., Christiansen C. (1987). Dietary calcium intake and blood pressure in normotensive subjects. Acta Medica Scand..

[107] Tomson J., Hin H., Emberson J., Kurien R., Lay M., Cox J., Hill M., Arnold L., Leeson P., Armitage L. (2017). Effects of Vitamin D on Blood Pressure, Arterial Stiffness, and Cardiac Function in Older People After 1 Year: BEST-D (Biochemical Efficacy and Safety Trial of Vitamin D). J. Am. Heart Assoc..

[108] Wamberg L., Kampmann U., Stødkilde-Jørgensen H., Rejnmark L., Pedersen S.B., Richelsen B. (2013). Effects of vitamin D supplementation on body fat accumulation, inflammation, and metabolic risk factors in obese adults with low vitamin D levels—Results from a randomized trial. Eur. J. Intern. Med..

[109] Wary C., Brillault-Salvat C., Bloch G., Leroy-Willig A., Roumenov D., Grognet J.M., Leclerc J.H., Carlier P.G. (1999). Effect of chronic magnesium supplementation on magnesium distribution in healthy volunteers evaluated by 31P-NMRS and ion selective electrodes. Br. J. Clin. Pharmacol..

[110] Whelton P.K., Buring J., Borhani N.O., Cohen J.D., Cook N., Cutler J.A., Kiley J.E., Kuller L.H., Satterfield S., Sacks F.M. (1995). The effect of potassium supplementation in persons with a high-normal blood pressure: Results from phase I of the Trials of Hypertension Prevention (TOHP). Ann. Epidemiol..

[111] Witham M.D., Adams F., Kabir G., Kennedy G., Belch J.J., Khan F. (2013). Effect of short-term vitamin D supplementation on markers of vascular health in South Asian women living in the UK--a randomised controlled trial. Atherosclerosis.

[112] Woo K.S., Yip T.W.C., Chook P., Koon K.V., Leong H.C., Feng X.H., Lee A.P.W., Kwok T.C.Y. (2021). Vitamins B-12 and C Supplementation Improves Arterial Reactivity and Structure in Passive Smokers: Implication in Prevention of Smoking-Related Atherosclerosis. J. Nutr. Health Aging.

[113] Wood A.D., Secombes K.R., Thies F., Aucott L., Black A.J., Mavroeidi A., Simpson W.G., Fraser W.D., Reid D.M., Macdonald H.M. (2012). Vitamin D3 supplementation has no effect on conventional cardiovascular risk factors: A parallel-group, double-blind, placebo-controlled RCT. J. Clin. Endocrinol. Metab..

[114] Yamamoto M.E., Applegate W.B., Klag M.J., Borhani N.O., Cohen J.D., Kirchner K.A., Lakatos E., Sacks F.M., Taylor J.O., Hennekens C.H. (1995). Lack of blood pressure effect with calcium and magnesium supplementation in adults with high-normal blood pressure: Results from phase I of the Trials of Hypertension Prevention (TOHP). Ann. Epidemiol..

[115] Chin S.F., Ibahim J., Makpol S., Abdul Hamid N.A., Abdul Latiff A., Zakaria Z., Mazlan M., Mohd Yusof Y.A., Abdul Karim A., Wan Ngah W.Z. (2011). Tocotrienol Rich Fraction Supplementation Improved Lipid Profile And Oxidative Status in Healthy Older Adults: A Randomized Controlled Study. Nutr. Metab..

[116] Entezari M.H. (2015). The Effect of Supplementary Calcium on Blood Pressure in Healthy Adult Women Aged 18-30 Years in Tehran, Iran. J. Educ. Health Promot..

[117] Goon J.A., Nor Azman N.H.E., Abdul Ghani S.M., Hamid Z., Wan Ngah W.Z. (2017). Comparing Palm Oil Tocotrienol Rich Fraction With A-Tocopherol Supplementation on Oxidative Stress in Healthy Older Adults. Clin. Nutr. ESPEN.

[118] Guerrero-Romero F., Rodríguez-Morán M. (2011). Magnesium Improves The Beta-Cell Function To Compensate Variation of Insulin Sensitivity: Double-Blind, Randomized Clinical Trial. Eur. J. Clin. Investig..

[119] Johnson N.E., Smith E.L., Freudenheim J.L. (1985). Effects on blood pressure of calcium supplementation of women. Am. J. Clin. Nutr..

[120] McSorley P.T., Bell P.M., Young I.S., Atkinson A.B., Sheridan B., Fee J.P.H., McCance D.R. (2005). Endothelial Function, Insulin Action And Cardiovascular Risk Factors in Young Healthy Adult Offspring of Parents With Type 2 Diabetes: Effect of Vitamin E in A Randomized Double-Blind, Controlled Clinical Trial. Diabet. Med..

[121] Miller J.Z., Weinberger M.H., Christian J.C. (1987). Blood Pressure Response to Potassium Supplementation in Normotensive Adults And Children. Hypertension.

[122] Moghassemi S., Marjani A. (2014). The Effect of Short-Term Vitamin D Supplementation on Lipid Profile and Blood Pressure In Post-Menopausal Women: A Randomized Controlled Trial. Iran. J. Nurs. Midwifery Res..

[123] Yanovski J.A., Parikh S.J., Yanoff L.B., Denkinger B.I., Calis K.A., Reynolds J.C., Sebring N.G., McHugh T. (2009). Effects of calcium supplementation on body weight and adiposity in overweight and obese adults: A randomized trial. Ann. Intern. Med..

[124] Zittermann A., Frisch S., Berthold H.K., Götting C., Kuhn J., Kleesiek K., Stehle P., Koertke H., Koerfer R. (2009). Vitamin D Supplementation Enhances The Beneficial Effects of Weight Loss on Cardiovascular Disease Risk Markers. Am. J. Clin. Nutr..

[125] Whelton P.K., He J., Appel L.J., Cutler J.A., Havas S., Kotchen T.A., Roccella E.J., Stout R., Vallbona C., Winston M.C. (2002). National High Blood Pressure Education Program Coordinating Committee. Primary prevention of hypertension: Clinical and public health advisory from The National High Blood Pressure Education Program. JAMA.

[126] Golzarand M., Shab-Bidar S., Koochakpoor G., Speakman J.R., Djafarian K. (2016). Effect of Vitamin D3 Supplementation on Blood Pressure In Adults: An Updated Meta-Analysis. Nutr. Metab. Cardiovasc. Dis..

[127] Beveridge L.A., Struthers A.D., Khan F., Jorde R., Scragg R., Macdonald H.M., Alvarez J.A., Boxer R.S., Dalbeni A., Gepner A.D. (2015). Effect of Vitamin D Supplementation on Blood Pressure: A Systematic Review and Meta-analysis Incorporating Individual Patient Data. JAMA Intern. Med..

[128] He S., Hao X. (2019). The effect of vitamin D3 on blood pressure in people with vitamin D deficiency: A system review and meta-analysis. Medicine.

[129] van Mierlo L.A.J., Arends L.R., Streppel M.T., Zeegers M.P.A., Kok F.J., Grobbee D.E., Geleijnse J.M. (2006). Blood pressure response to calcium supplementation: A meta-analysis of randomized controlled trials. J. Hum. Hypertens..

[130] Morvaridzadeh M., Sepidarkish M., Fazelian S., Rahimlou M., Omidi A., Ardehali S.H., Sanoobar M., Heshmati J. (2020). Effect of Calcium and Vitamin D Co-supplementation on Blood Pressure: A Systematic Review and Meta-Analysis. Clin. Ther..

[131] Rosanoff A., Costello R.B., Johnson G.H. (2021). Effectively Prescribing Oral Magnesium Therapy for Hypertension: A Categorized Systematic Review of 49 Clinical Trials. Nutrients.

[132] Poorolajal J., Zeraati F., Soltanian A.R., Sheikh V., Hooshmand E., Maleki A. (2017). Oral potassium supplementation for management of essential hypertension: A meta-analysis of randomized controlled trials. PLoS ONE.

[133] Godswill A.G., Somtochukwu I.V., Ikechukwu A.O., Kate E.C. (2020). Health benefits of micronutrients (vitamins and minerals) and their Associated Deficiency Diseases: A systematic review. Int. J. Food Sci..

[134] Whelton P.K., Carey R.M., Mancia G., Kreutz R., Bundy J.D., Williams B. (2022). Harmonization of the American College of Cardiology/American Heart Association and European Society of Cardiology/European Society of Hypertension Blood Pressure/Hypertension Guidelines: Comparisons, Reflections, and Recommendations. Circulation.

[135] Unger T., Borghi C., Charchar F., Khan N.A., Poulter N.R., Prabhakaran D., Ramirez A., Schlaich M., Stergiou G.S., Tomaszewski M. (2020). 2020 International Society of Hypertension Global Hypertension Practice Guidelines. Hypertension.

