# Supplementary material for: Vitamins and Minerals for Blood Pressure Reduction in the General, Normotensive Population: A Systematic Review and Meta-Analysis of Six Supplements
Source: Nutrients. 2023 Sep 30;15(19):4223. doi: 10.3390/nu15194223 (PMC10574336; doi:10.3390/nu15194223)
Supplement: Supplementary file 1 [file nutrients-15-04223-s001.zip › nutrients-2630265-supplementary.pdf]

Supplementary Data File

Table S1: Search terms for each of the four databases.

Cochrane<br>Date run: 7/22/2022<br>Number of results: 4,497	(([mh "ascorbic acid"] OR [mh "vitamin D"] OR [mh "vitamin E"] OR [mh "Vitamin B Complex"] OR "vitamin C":ti,ab OR "ascorbic acid":ti,ab OR "vitamin D":ti,ab OR "vitamin E":ti,ab OR "vitamin B":ti,ab OR ("B" NEXT vitamin*):ti,ab OR [mh calcium] OR [mh magnesium] OR [mh potassium] OR calcium:ti,ab OR magnesium:ti,ab OR potassium:ti,ab) AND ([mh "blood pressure"] OR "blood pressure":ti,ab)) AND ("Randomized controlled trial":pt OR "controlled clinical trial":pt)
Embase<br>Date run: 7/22/2022<br>Number of results: 6,585	(('ascorbic acid'/exp OR 'vitamin D'/exp OR 'vitamin E'/exp OR 'Vitamin B Complex'/exp OR 'vitamin C':ti,ab OR 'ascorbic acid':ti,ab OR 'vitamin D':ti,ab OR 'vitamin E':ti,ab OR 'vitamin B':ti,ab OR 'B vitamin*':ti,ab OR calcium/exp OR magnesium/exp OR potassium/exp OR calcium:ti,ab OR magnesium:ti,ab OR potassium:ti,ab) AND ('blood pressure'/exp OR 'blood pressure':ti,ab)) AND (‘randomized controlled trial’ OR ‘controlled clinical trial’)
MEDLINE (PubMed)<br>Date run: 7/22/2022<br>Number of results: 4,579	((ascorbic acid[MeSH] OR vitamin D[MeSH] OR vitamin E[MeSH] OR Vitamin B Complex[MeSH] OR “vitamin C”[tiab] OR “ascorbic acid”[tiab] OR “vitamin D”[tiab] OR “vitamin E”[tiab] OR “vitamin B”[tiab] OR B vitamin*[tiab] OR calcium[MeSH] OR magnesium[MeSH] OR potassium[MeSH] OR calcium[tiab] OR magnesium[tiab] OR potassium[tiab]) AND (blood pressure[MeSH] OR “blood pressure”[tiab])) AND (randomized controlled trial[pt] OR controlled clinical trial[pt])
Web of Science (Core Collection)<br>Date run: 7/22/2022<br>Number of results: 537	((TI="vitamin C" OR AB="vitamin C") OR (TI="ascorbic acid" OR AB="ascorbic acid") OR (TI="vitamin D" OR AB="vitamin D") OR (TI="vitamin E" OR AB="vitamin E") OR (TI="vitamin B" OR AB="vitamin B") OR (TI="B vitamin*" OR AB="B vitamin*") OR (TI=calcium OR AB=calcium) OR (TI=magnesium OR AB=magnesium) OR (TI=potassium OR AB=potassium) AND (TI="blood pressure" OR AB="blood pressure")) AND (ALL="randomized controlled trial" OR ALL="controlled clinical trial")

Table S2: Reasons for exclusion of full-text articles (n = 325).

Number of Studies Excluded	Reason
159	Wrong patient population (i.e., hypertensive at baseline or ≥50% of participants share a common co-morbidity)
26	Wrong study design (i.e., not a RCT)
22	Dietary changes, not supplement
22	Less than two weeks in duration
22	Duplicate
21	No placebo
20	Wrong outcomes (i.e., no BP data)
13	Multiple supplements used in combination
6	Wrong intervention
3	Abstract only
2	Full-article not accessible
2	Study not in English
2	Pediatric population
2	Protocol for a RCT only
2	Wrong route of administration
1	Fortified food

Table S3: Reasons for exclusion of the five studies that met eligibility.

Study	Reason
Luft 1989 [68] and Mullen 1990 [80]	They are both crossover trials. Researchers usually would do the paired analysis, but they are not often published. The data we have are mean and standard deviation for intervention and control separately. Cochrane offered three potential solutions: (1) Treat them as a parallel analysis, however, not recommended; (2) incorporating them to include data from the first period; however, the first period result wasn't reported; (3) incorporating inappropriately reported cross-over trials is to attempt to approximate a paired analysis, by imputing missing standard deviations. We performed solution (3) for all the cross-over trials included in this study because the others have only one active intervention, which is the case in which this method was proposed. For these two studies with more than one active interventions, if we did method (3) or method (1), it could possibly introduce bias because of the carry-over effect.
Mostafa 1989 [77]	There was no data provided for the placebo group, so this study was unable to be used in a pairwise meta-analysis.
Mottram 1999 [78]	We could calculate systolic and diastolic BP from the given MAP, but not the SDs. To calculate the SDs, we would need to assume a correlation coefficient between MAP and PP, then assume a correlation coefficient between SBP and DBP. Later, we still need to assume another correlation between baseline and final to calculate MD. We deemed this to be too many assumptions with a risk of introducing bias.
Nowson 1989 [84]	This study had the change from baseline data for normotensives but lacked the corresponding SDs, as well as the baseline and final BP values. Therefore, we determined the only solution to imputing these SDs was to find a similar paper with the same intervention, treatment duration, and number of participants and use the SDs from this study for imputation. We were unable to find a study with these matching characteristics.

Table S4: Basic characteristics of included studies.

Study (Last Name, Year)	Population	Type of Trial	Trial Arm	Dosage	Sample Size	Baseline BP (Systolic/ Diastolic)	Mean Age	Length of Trial	Adverse Events/ Side Effects
Agbalalah 2022 [35]	Healthy Overweight (BMI ≥ 24.9)	Parallel	Vitamin D3	5000 IU/d	24	128.7/77	35.9	2 months	
			Placebo	-	24	131.2/78	33.1	2 months	
Al-Dujaili 2016 [34]	Healthy adults ages 19-53	Parallel	Placebo	-	6	116.7/73/7	25.2	0.5 months	
			Vitamin D3	2000 IU/d	9	115.8/75.4	27.75	0.5 months	
Aringazina 2021 [35]	Healthy adults ages 50-60	Parallel	Placebo	-	150	126.1	56.27	4 years	
			Vitamin D3	2000 IU/d	186	125.95	56.27	4 years	
Barden 1986 [36]	Healthy normotensive adult females ages 18-55	Cross-over	Placebo	-	22	120.5/73.6	30.95	4 weeks	
			Potassium	80 mmol/d	22	114.5/69.2	32.12	4 weeks	
Belizan 1983 [37]	Healthy young adults ages 18-35	Parallel	Placebo	-	27	104.4/67.8	24.7	22 weeks	
			Calcium	1000 mg/d	30	110.95/71.6	24.85	22 weeks	
Berry 2010 [38]	Adults ages 22-65 with high-normal/elevated BP	Cross-over	Placebo	-	48	137.6/88.6	45.14	6 weeks	
			Potassium citrate	40 mmol/d	48	137.6/88.6	45.14	6 weeks	
Bostick 2000 [39]	Adults ages 30-74 who had been diagnosed as having adenomatous polyps within the previous 5 years (35% HTN; 36% hyperlipidemia; 58% of women were postmenopausal; 20% smokers)	Parallel	Placebo	-	66	130.3	59	6 months	
			Calcium	1 g twice daily	64	130.1	60	6 months	
			Calcium	2 g twice daily	63	130.1	58	6 months	
Brancati 1996 [40]	Healthy African American adults ages 27-65 years old (18.2% smokers)	Parallel	Placebo	-	44	126.9/77.7	49.6	21 days	
			Potassium	80 mmol/d	43	125.4/77.4	46.4	21 days	
		Parallel	Placebo	-	42	107.84/66.33	33.8	6 weeks	

Braschi 2008 [41]	Adults ages 22 to 65 (44.1% with familial HTN)<br>Adults ages 22 to 65 (30% with familial HTN)<br>Adults ages 22 to 65 (38.5% with familial HTN)		Potassium citrate	30 mmol/d	38	114.67/70.2	36.2	6 weeks	
			Potassium chloride	30 mmol/d	34	111.88/68.49	36.9	6 weeks	
Bressendorff 2016 [42]	Healthy white adults	Parallel	Placebo	-	18	118/73	44.5	4 months	
			Cholecalciferol (Vitamin D)	3000 IU/d	22	117.7/72.1	41	4 months	
Chai 2013 [43]	Adults in good health with an adenomatous colorectal polyp within the past 36 months (82% obese, 48% HTN, 45% HLD)	Parallel	Placebo	-	23	123.3/80.5	58.5	6 months	
			Calcium	1 g twice daily	23	125.2/79.1	61.9	6 months	
			Vitamin D3	400 IU twice daily	23	125.9/76.5	60.2	6 months	
Cheung 2022 [44]	Adults who are overweight/obese	Parallel	Placebo	-	23	117.6/80.9	41	3 months	
			Vitamin D3	1000 IU D3 3x daily	34	120.5/78.4	43.5	3 months	
Chin 2011 [115]	Two groups:<br>1. Healthy adult non-smoking females 35-49 y/o<br>2. Healthy adult non-smoking females over 50 y/o	Parallel	Vitamin E (1)	160 mg/day	16	121/80.6	44.5	6 months	
			Vitamin E (2)	160 mg/day	16	131/81.3	56.1	6 months	
			Placebo (1)	-	15	121/75.4	44.7	6 months	
			Placebo (2)	-	15	137/82	59.1	6 months	
Cosaro 2014 [45]	Healthy participants with normomagnesemia ages 23-33	Cross-over	Placebo	-	8	122.7/72.1	26.3	8 weeks	
			Magnesium-pidolate	8.1 mmol twice daily	6	126/71.4	26.3	8 weeks	
Doyle 1999 [46]	Healthy normotensive young adult females ages 20-28	Cross-over	Placebo	-	12	Not given	23	28 days	
			Magnesium hydroxide	10 mmol/d	13	Not given	23	28 days	
Dreier 2020 [47]	Healthy normotensive men ages 20-55	Cross-over	Placebo	-	12	119.7/72.6	25.7	4 weeks	
			Potassium	90 mmol/d	13	119.7/72.6	25.7	4 weeks	
		Parallel	Calcium	1000 mg/day	27	116.5/77.1	22.6	1 month	

Entezari 2015 [116]	Normotensive women ages 18-30 with BMI < 27		Placebo	-	26	114/73.1	23.4	1 month	
Finstad 2001 [48]	Physically active women ages 17 to 43	Cross-over	Placebo	-	19	113/69	21.4	4 weeks	
			Magnesium oxide	212 mg/d	13	116.3/70.2	20.8	4 weeks	
Forman 2013 [49]	Black adults (41.7% taking BP meds)	Parallel	Placebo	-	72	120/78	51	6 months	
			Cholecalciferol	1000 IU/d	68	123/80	51	6 months	
			Cholecalciferol	2000 IU/d	73	121/75	50	6 months	
			Cholecalciferol	4000 IU/d	70	128/78	51	6 months	
Forouhi 2016 [50]	Adults at risk of type 2 diabetes	Parallel	Placebo	-	114	127.9/77.6	52.4	4 months	
			Vitamin D2	100,000 IU/month	112	126.9/75.8	53.5	4 months	
			Vitamin D3	100,000 IU/month	114	128.6/77.6	52.5	4 months	
Fotherby 2000 [51]	Adults ages 60 to 80 never having received treatment for HTN	Cross-over	Placebo	-	23	136/79	72	3 months	
			Vitamin C	500 mg/d	17	134/78	72	3 months	
Gariballa 2022 [52]	Arabian adults aged 18+	Parallel	Placebo	-	66	122/77	41	6 months	
			Vitamin D3	2000 IU/d	68	118/75	42	6 months	
			Calcium	600 mg/d	75	116/76	41	6 months	
Goon 2017 [117]	Healthy adults aged 50-55 y/o	Parallel	Vitamin E (tocotrienol rich fraction)	150 mg/day	24	130.6/80	53.4	6 months	
			Vitamin E (alpha-tocopherol)	400 IU/day	24	131.1/81.3	52.5	6 months	
			placebo	-	23	123.5/78.3	52.2	6 months	
Gu 2001 [53]	Adults ages 35-64 from Beijing, China with high normal BP or mild HTN, but never receiving treatment	Parallel	Placebo	-	75	134/83	55	3 months	
			Potassium chloride	60 mmol/day	75	136.9/81.5	56.9	3 months	
		Parallel	Placebo	-	31	111/73	42.2	3 months	

Guerrero-Romero 2004 [54]	Healthy adults with insulin resistance (HOMA-IR index equal or greater than 3.0) and hypomagnesemia (Serum magnesium levels equal or lower than 0.74 mmol/l)		Magnesium chloride	2.5 g/d	32	110/73	43	3 months	
Guerrero-Romero 2011 [118]	non-diabetic normotensive adults	Parallel	5 % MgCl2	50 mL/day	49	117.2/73.8	39.8	3 months	
			placebo	-	48	115.9/73.8	41.4	3 months	
Hofmeyr 2021 [55]	Non-pregnant women who had pre-eclampsia or eclampsia in their most recent pregnancy	Parallel	Placebo	-	404	127.6/82.7	30.9	3 months	
			Calcium	500 mg/d	387	128.6/82.8	30.5	3 months	
Hutchins 2005 [56]	Healthy, nonsmoking, postmenopausal women	Cross-over	Placebo	-	8 women total	117/70	56	3.5 months	
			Vitamin C	500 mg		119/69	56	3.5 months	
Indhavivahdana 2022 [57]	Menopausal Thai women ages 40-60 without known cardio-metabolic disease, kidney disease, etc.	Parallel	Placebo	-	37	126/74	53.19	3 months	
			Vitamin D2	20,000 IU twice a week	39	119/73.11	54.21	3 months	
Itoh 1997 [58]	Healthy Japanese adults	Parallel	Placebo	-	10	121/74	66	1 month	
			Magensium hydroxide	411-548 mg/d	23	130/77	64	1 month	
Johnson 1985 [119]	35-65 y/o normotensive women	Parallel	Calcium	1.5 g/day	44	119/73	52	4 years	
			placebo	-	51	120/75	54.6	4 years	
Joris 2016 [59]	Overweight and obese adults	Parallel	Placebo	-	25	126/81	62	6 months	
			Magnesium	117 mg three times per day	26	130/82	62	6 months	
Kass 2015 [60]	Adults recruited from recreational running, cycling and triathlete	Cross-over	Placebo	-	3	119/85	40.8	4 weeks	
			Magnesium	300 mg/d	4	118/79	40.8	4 weeks	

	clubs given a chronic intervention								
Keith 1982 [61]	Nonsmokers ages 25-38	Cross-over	Placebo	-	10	123/75	28.4	3 weeks	
			Ascorbic acid (Vitamin C)	300 mg/d	10	127/74	28.4	3 weeks	
Khaw 1982 [62]	Young healthy males ages 22-35	Cross-over	Placebo	-	10	118/73.5	N/G	1 month	
			Oral potassium	64 mmol/d	10	118/73.5	N/G	1 month	
Khosravi 2018 [63]	Overweight and obese women ages 20-40	Parallel	Placebo	-	27	112.5/78.5	26.9	1.5 months	
			Vitamin D	50,000 IU/w	26	112.7/80	29.1	1.5 months	
Kubiak 2018 [64]	Adults over 40 in Northern Norway with vitamin D deficiency	Parallel	Placebo	-	203	123/77	51	4 months	
			Vitamin D	100,000 IU loading dose, then 20,000 IU/w	208	122/77	50	4 months	
Lee 2009 [65]	Nondiabetic overweight Korean adults with normomagnesemia	Parallel	Placebo	-	80	126.7/83.3	40.5	3 months	
			Magnesium oxide	12.3 mmol/d	75	124.7/83.5	39.6	3 months	
Li 2010 [66]	Obese Chinese women not taking HTN or HLD meds and no hx of MI or diabetes	Parallel	Placebo	-	29	129.5/84.7	41.2	6.5 months	
			Calcium	162 mg/day	28	129/85.2	41.6	6.5 months	
Lijnen 1995 [67]	Normal male adults	Parallel	Placebo	-	16	114/72	24.4	4 months	
			Oral calcium	1g twice a day	16	114/74	24.2	4 months	
Lutsey 2018 [69]	Adults 55+ y/o; exclusion criteria included a prior history of heart disease, stroke, or kidney disease; the use of type I or III antiarrhythmic drugs or digoxin; the current use of magnesium supplements; any prior history of allergy or intolerance to	Parallel	Placebo	-	30	119/71	61.6	3 months	
			Magnesium	400 mg/d	29	119/72	61.3	3 months	

	magnesium; lactose intolerance; and a prior history of inflammatory bowel disease or any severe gastrointestinal disorder.								
Lyle 1987 [70]	Healthy White men ages 19-52	Parallel	Placebo	-	27	114.9/74.7	34.8	3 months	
			Calcium	1500 mg/d	27	115.8/74.3	31.3	3 months	
	Healthy Black men ages 19-52	Parallel	Placebo	-	11	113.5/71.0	28.3	3 months	
			Calcium	1500 mg/d	10	114.7/70.2	28.2	3 months	
Lyle 1992 [71]	Healthy adults with high normal to mildly hypertensive BP	Parallel	Calcium	1500 mg/d	21	132.4/88.2	32.3	2 months	
			Placebo	-	21	132.7/86.5	35.9	2 months	
Matthesen 2012 [72]	Healthy white adults ages 18-40	Cross-over	Placebo	-	21	116/71	26	28 days	
			Potassium	100 mmol/d	21	116/71	26	28 days t	
McCarron 1985 [73]	Healthy normotensive adults ages 21-70	Cross-over	Placebo	-	32	113/75	47.7	8 weeks	
			Calcium	1000 mg/d	32	113/75	47.7	8 weeks	
McMullan 2017 [74]	Normotensive overweight or obese adults with vitamin D deficiency	Parallel	Placebo	-	27	123.7/73.7	34.7	2 months	abdominal discomfort, dry mouth, elevated serum phosphorous
			Vitamin D	50,000 IU/week	29	120.4/71.6	39.3	2 months	abdominal discomfort, elevated potassium
McSorley 2005 [120]	Healthy adults with Type 2 Diabetic Parents	Cross-over	Vitamin E	800 IU/day	13	SBP: 118	28	3 months	
			Placebo	-	13	SBP: 118	28	3 months	
Miller 1987 [121]	General adult population	Parallel	Potassium	53.7 mEq/day for women 66.0 mEq/day for men	64	113.2/73.1	42	1 month	
			Placebo	-	64	113.2/73.1	42	1 month	
		Parallel	Placebo	-	50	113/71	29	3 months	

Mitchell 2015 [75]	Healthy adults ages 18-45		Vitamin D	50,000 IU/week	40	114/72	28	3 months	
Moghassemi 2014 [122]	Post-menopausal women with Vitamin D insufficiency	Parallel	Vitamin D3	2000 IU/day	38	126.9/77.7	52.7	3 months	
			Placebo	-	38	122.9/76.9	51.9	3 months	
Mooren 2011 [76]	Overweight, insulin resistant, non-diabetic adults ages 30-70	Parallel	Placebo	-	22	134.8/82.5	N/G	6 months	
			Magnesium	365 mg/d	25	137.7/85.3	N/G	6 months	
Muldowney 2012 [79]	General population ages 20-40	Parallel	Placebo	-	56	124/75	N/G	22 weeks	
			Vitamin D	200 IU/d	44	123/73	N/G	22 weeks	
			Vitamin D	400 IU/d	51	126/76	N/G	22 weeks	
			Vitamin D	600 IU/d	51	124/77	N/G	22 weeks	
Nagpal 2009 [81]	Healthy, centrally obese males older than 35 y/o	Parallel	Placebo	-	36	124/77	45	6 weeks	
			Vitamin D3	120,000 IU/biweekly	35	124/78	42.4	6 weeks	
Naismith 2003 [82]	General population ages 25-65 (28% smokers, 7% HTN)	Parallel	Placebo		29	115.7/70.5	41.7	6 weeks	
			Potassium Chloride	24 mmol/d	30	118.2/75.5	44.5	6 weeks	
Nimitphong 2015 [83]	Adults ages 35-80 with impaired fasting glucose and/or impaired glucose tolerance	Parallel	Placebo	-	18	127.2/82.6	57.9	3 months	
			Vitamin D2	20,000 IU/week	19	126.9/76.9	61.2	3 months	
			Vitamin D3	15,000 IU/week	10	126.9/76.9	63	3 months	
Osilesi 1991 [85]	Healthy adults ages 28-77 (hypertensive subjects excluded)	Cross-over	Placebo	-	8	Not given	57.8	6 weeks	
			Vitamin C	1000 mg/d	8	Not given	57.8	6 weeks	
Patil 2009 [86]	Healthy cyclists ages 18-19	Parallel	Vitamin E	200 mg/d	19	121.05/76.84	N/G	21 days	
			Placebo	-	18	117.0/76.77	N/G	21 days	
Ramly 2014 [87]	Postmenopausal women	Parallel	Placebo	-	99	118.9/76.79	42.88	12 months	
			Vitamin D	50,000 IU/week for 2 months, then 50,000 IU/month for 10 months	93	121.6/77.77	42.58	12 months	

Rasool 2003 [88]	Postmenopausal women	Cross-over	Placebo	-	17	121/72	54.59	10 weeks	
			Vitamin E	400 IU/d	17	121/72	54.59	10 weeks	
Rasool 2006 [89]	Healthy men (between 21-30 years old)	Parallel	Placebo	-	9	SBP: 104.9	N/G	2 months	
			Vitamin E	80 mg/d	9	SBP: 97.6	N/G	2 months	
			Vitamin E	160 mg/d	9	SBP: 103.6	N/G	2 months	
			Vitamin E	320 mg/d	9	SBP: 101.9	N/G	2 months	
Rasool 2008 [90]	Healthy men (under 40 years old)	Parallel	Placebo	-	9	125.2/75.8	24.1	2 months	
			Vitamin E	50 mg/d	9	122.2/74.1	24.1	2 months	
			Vitamin E	100 mg/d	9	120.9/75.0	23.8	2 months	
			Vitamin E	200 mg/d	9	124.6/76.4	23.4	2 months	
Reid 2005 [91]	Postmenopausal women	Parallel	Placebo	-	739	133.9/69.6	74.3	30 months	
			Calcium	1 g/d	732	134.9/70.1	74.2	30 months	
Reid 2010 [92]	Healthy men	Parallel	Placebo	-	107	129.7/78.3	57	24 months	
			Calcium	600 mg/d	108	130.7/78.1	55	24 months	
			Calcium	1200 mg/d	108	132.3/78.9	57	24 months	
Rodríguez-Moran 2014 [93]	Healthy adults with hypomagnesemia	Parallel	Placebo	-	23	112.3/71.4	39.5	4 months	
			Magnesium Chloride	382 mg/d	24	111.3/71.5	31.9	4 months	Mild diarrhea
Rodríguez-Ramírez 2017 [94]	Pre-hypertensive adults ages 35-65 with hypomagnesemia	Parallel	Placebo	-	18	126/75.6		4 months	
			Magnesium Lactate	360 mg/d	18	129.2/78.9		4 months	Mild diarrhea
Sacks 1998 [95]	Healthy women	Parallel	Placebo	-	102	115/73	38	4 months	
			Potassium Chloride	40 mmol/d	46	118/75	39	4 months	
			Calcium Carbonate	1200 mg/d	51	117/74	39	4 months	
			Magnesium Lactate	336 mg/d	48	116/73	39	4 months	

Sakai 2016 [96]	Postmenopausal women	Parallel	Placebo	-	15	137.6/83.7	61.7	12 months	
			Calcium Carbonate	300 mg/d	14	131.4/83.3	60.9	12 months	
Salehpour 2011 [97]	Healthy premenopausal overweight +obese women	Parallel	Placebo	-	38	116.7/71.9	37	3 months	
			Vitamin D3	25 µg/d	39	110.5/67.9	38	3 months	
Schutten 2022 [98]	Overweight + Slightly Obese adults ages 45-75 (BMI 25-35 kg/m2)	Parallel	Placebo	-	26	130/80	63.8	6 months	
			Magnesium Citrate	450 mg/d	46	133/79	64.1	6 months	flatulence, stomach pain, diarrhea
			Magnesium Oxide	450 mg/d	46	127/77	63.2	6 months	
			Magnesium Sulfate	450 mg/d	46	130/79	62	6 months	Not related to intervention (stroke)
Scragg 2014 [99]	Healthy adults	Parallel	Placebo	-	151	123.4/76.3	47.8	18 months	
			Vitamin D3	200,000 IU/month for 2 months 100,000 IU/month for remaining 16 months	149	122.6/75.6	47.4	18 months	
Seibert 2017 [100] Seibert 2017	Healthy adults ages 20-71	Parallel	Placebo	-	51	112/75	39	3 months	
			Vitamin D3	20 µg/d	54	113/73	39	3 months	
Shidfar 2011 [101]	Overweight men	Parallel	Placebo	-	24	121.1/80.9	33.8	2 months	
			Calcium Carbonate	1250 mg/d	25	116.8/79.5	35.1	2 months	
Shockravi 2008 [102]	Overweight/obese women	Parallel	Placebo	-	20	99/68	25.4	1 month	
			Calcium Carbonate	1000 mg/d	24	97/66	24.5	1 month	
		Parallel	Placebo	-	28	115.7/72.3	41.1	3 months	

Simental-Mendia 2014 [103]	Prediabetic. hypomagnesia adults ages 18-65		Magnesium Chloride	382 mg/d	29	114.8/76.9	39.8	3 months	
Sluyter 2017 [104]	Older adults ages 50-84 (44% on HTN meds)	Parallel	Placebo	-	261	137.7/78.4	65.5	1.1 years	
			Vitamin D3	200,000 IU loading dose 100,000 IU monthly	256	137.7/78.7	64.5	1.1 years	
Sollid 2014 [105]	Prediabetic adults	Parallel	Placebo	-	242	135.8/82.8	61.9	12 months	
			Vitamin D3	20,000 IU weekly	242	135.1/83.6	62.3	12 months	
Thomsen 1987 [106]	Postmenopausal women	Parallel	Placebo	-	14	122.3/76.8	N/G	12 months	
			Calcium	2000 mg/d	14	125.9/75.3	N/G	12 months	
Tomson 2017 [107]	Older adults age 65+ (39% with HTN) (General)	Parallel	Placebo	-	95	129.5/76.6	72	12 months	
			Vitamin D	2000 IU/d	98	131.8/76.6	72	12 months	
			Vitamin D	4000 IU/d	97	132.7/78	71	12 months	
Wamberg 2013 [108]	Healthy adults with BMI > 30 kg/m2 (Obese)	Parallel	Placebo	-	21	132/82	41.2	26 weeks	constipation, nausea, tiredness, headaches
			Vitamin D	7000 IU/d	22	133/84	39.5	26 weeks	constipation, nausea, tiredness, headaches
Wary 1999 [109]	Healthy young males	Parallel	Placebo		15	126/77	23.7	1 month	
			Magnesium	12 mmol/d	15	127/76	23.7	1 month	
Whelton 1995 [110]	Healthy adults	Parallel	Placebo	-	175	122.6/81.1	43.1	6 months	
			Potassium Chloride	60 mmol/d	178	120.7/80.8	42.8	6 months	
Witham 2013 [111]	Healthy South Asian females living in the UK	Parallel	Placebo	-	23	122/78	39.4	1 month	11 minor events- headache, constipation,

									vomiting, diarrhea, skin rash, corneal ulcer, menorrhagia, chest pain, insomnia
			Vitamin D3	100,000 IU single dose	25	119/78	41.7	1 month	8 events- headache, diarrhea, constipation, UTI, joint pain, subconjunctival hemorrhage
Woo 2021 [112]	Healthy but exposed to secondhand smoke (casino workers)	Parallel	Placebo	-	21	123/83	45	12 months	
			Vitamin C	200 mg/d	19	117/78	45	12 months	
Wood 2012 [113]	Postmenopausal women ages 60-70	Parallel	Placebo	-	98	128.18/77.7	63.9	12 months	
			Vitamin D3	400 IU/d	96	128.16/77.68	63.5	12 months	
			Vitamin D3	1000 IU/d	95	129.15/76.96	64.1	12 months	
Yamamoto 1995 [114]	Healthy population ages 30-54	Parallel	Placebo	-	234	125.4/83.9	42.4	6 months	
			Calcium	1 g/d	237	126.0/84.1	42.7	6 months	
			Magnesium	360 mg/d	227	124.2/83.7	42.7	6 months	
Yanovski 2009 [123]	Healthy overweight (BMI ≥ 25)	Parallel	Calcium	1500 mg/day	168	Not given	38.9	24 months	
			Placebo	-	167	Not given	38.7	24 months	
Zitterman 2009 [124]	Healthy overweight (BMI > 27)	Parallel	Vitamin D	3332 IU/daily	82	128/86	47.4	12 months	
			Placebo	-	83	128/86	48.8	12 months	

Including population, type of trial, length of trial, and arms of the trial with dosage, sample size, baseline BP, mean age, and whether there were any adverse events or side effects. If no adverse events or side effects were reported, the corresponding part of the table was left blank. N/G in the mean age column stands for “Not given.”

Table S5: Risk of bias results by individual study.

Study (87)	Domain 1	Domain 2	Domain 3	Domain 4	Domain 5	Overall
Agbalalah 2022 [33]	Low	Low	Low	Low	Low	Low
Al-Dujaili 2016 [34]	Low	Some concerns	Low	Some concerns	Low	Some concerns
Aringazina 2021 [35]	Some concerns	Some concerns	Low	Some concerns	Low	High
Barden 1986 [36]	Low	Some concerns	Low	Some concerns	Low	Some concerns
Belizan 1983 [37]	Low	Low	Some concerns	Low	Low	Low
Berry 2010 [38]	Low	Low	Low	Low	Low	Low
Bostick 2000 [39]	Low	Low	Low	Low	Low	Low
Brancati 1996 [40]	Low	Low	Low	Low	Low	Low
Braschi 2008 [41]	Low	Low	Low	Low	Low	Low
Bressendorff 2016 [42]	Low	Low	Some concerns	Low	Low	Low
Chai 2013 [43]	Low	Low	Low	Low	Low	Low
Cheung 2022 [44]	Low	Low	Low	Low	Low	Low
Chin 2011 [115]	Low	Low	Low	Low	Low	Low
Cosaro 2014 [45]	Low	Low	Low	Low	Low	Low
Doyle 1999 [46]	Low	Low	Low	Low	Low	Low
Dreier 2020 [47]	Low	Low	Low	Low	Low	Low
Entezari 2015 [116]	Some concerns	Some concerns	High	Low	Low	High
Finstad 2001 [48]	Low	Low	Some concerns	Low	Low	Low
Forman 2013 [49]	Low	Low	Low	Low	Low	Low
Forouhi 2016 [50]	Low	Low	Low	Low	Low	Low
Fotherby 2000 [51]	Low	Low	Low	Low	Low	Low
Gariballa 2022 [52]	Low	Low	Some concerns	Low	Low	Low

Goon 2017 [117]	Low	Low	Low	Low	Low	Low
Gu 2001 [53]	Low	Some concerns	Low	Low	Low	Low
Guerrero-Romero 2004 [54]	Low	Low	Low	Low	Low	Low
Guerrero-Romero 2011 [118]	Low	Low	Low	Low	Low	Low
Hofmeyr 2021 [55]	Low	Low	Low	Low	Low	Low
Hutchins 2005 [56]	Low	Some concerns	Low	Low	Low	Low
Indhavivadhana 2022 [57]	Low	Low	Low	Low	Low	Low
Itoh 1997 [58]	Low	Low	Low	Low	Low	Low
Johnson 1985 [119]	Some concerns	Some concerns	Low	Low	Low	Some concerns
Joris 2016 [59]	Low	Low	Low	Low	Low	Low
Kass 2015 [60]	Low	Some concerns	Low	Low	Low	Low
Keith 1982 [61]	Low	Low	Low	Low	Low	Low
Khaw 1982 [62]	Some concerns	Low	Low	Low	Low	Low
Khosravi 2018 [63]	Low	Low	Some concerns	Low	Low	Low
Kubiak 2018 [64]	Low	Low	Low	Low	Low	Low
Lee 2009 [65]	Low	Low	Low	Low	Low	Low
Li 2010 [66]	Low	Low	Low	Low	Low	Low
Lijnen 1995 [67]	Low	Some concerns	Low	Low	Low	Low
Lutsey 2018 [69]	Low	Low	Low	Low	Low	Low
Lyle 1987 [70]	Low	Low	Low	Low	Low	Low
Lyle 1992 [71]	Low	Low	Low	Low	Low	Low
Matthesen 2012 [72]	Low	Some concerns	Some concerns	Low	Low	Some concerns
McCarron 1985 [73]	Low	Low	Low	Low	Low	Low
McMullan 2017 [74]	Low	Low	Low	Low	Low	Low

McSorley 2005 [120]	Low	Low	Low	Low	Low	Low
Miller 1987 [121]	Some concerns	Some concerns	Low	Some concerns	Low	High
Mitchell 2015 [75]	Low	Low	Low	Low	Low	Low
Moghassemi 2014 [122]	Low	Low	Low	Low	Low	Low
Mooren 2011 [76]	Low	Some concerns	Low	Low	Low	Low
Muldowney 2012 [79]	Low	Low	Low	Low	Low	Low
Napgal 2009 [81]	Low	Low	Low	Low	Low	Low
Naismith 2003 [82]	Low	Low	Low	Low	Low	Low
Nimitphong 2015 [83]	Some concerns	Some concerns	Low	Low	Low	Some concerns
Osilesi 1991 [85]	Some concerns	Low	Low	Low	Low	Low
Patil 2009 [86]	Some concerns	Low	Low	Low	Low	Low
Ramly 2014 [87]	Low	Low	Low	Low	Low	Low
Rasool 2003 [88]	Low	Low	Low	Low	Low	Low
Rasool 2006 [89]	Some concerns	Some concerns	Low	Some concerns	Low	High
Rasool 2008 [90]	Some concerns	Low	Low	Low	Low	Low
Reid 2005 [91]	Low	Some concerns	Low	Some concerns	Low	Some concerns
Reid 2010 [92]	Low	Low	Low	Low	Low	Low
Rodriguez-Moran 2014 [93]	Low	Some concerns	Low	Low	Low	Low
Rodriguez-Ramirez 2017 [94]	Low	Some concerns	Low	Low	Low	Low
Sacks 1998 [95]	Some concerns	Low	Low	Low	Low	Low
Sakai 2017 [96]	Some concerns	High	Low	Low	Low	High

Salehpour 2012 [97]	Some concerns	Some concerns	Low	Low	Low	Some concerns
Schutten 2022 [98]	Low	Low	Low	Low	Low	Low
Scragg 2014 [99]	Low	Low	Low	Low	Low	Low
Seibert 2017 [100]	Low	Low	Low	Low	Low	Low
Shidfar 2011 [101]	Low	Some concerns	Low	Low	Low	Low
Shockravi 2008 [102]	Some concerns	Low	Low	Low	Low	Low
Simental-Mendia 2014 [103]	Some concerns	Some concerns	Low	Low	Low	Some concerns
Sluyter 2017 [104]	Low	Some concerns	Low	Low	Low	Low
Sollid 2014 [105]	Low	Low	Low	Low	Low	Low
Thomsen 1987 [106]	Low	Low	Low	Some concerns	Low	Low
Tomson 2017 [107]	Low	Low	Low	Low	Low	Low
Wamberg 2013 [108]	Low	Low	Low	Low	Low	Low
Wary 1999 [109]	Some concerns	Low	Low	Low	Low	Low
Whelton 1995 [110]	Low	Low	Low	Low	Low	Low
Witham 2013 [111]	Low	Low	Low	Low	Low	Low
Woo 2021 [[112]	Some concerns	Some concerns	Low	Low	Low	Some concerns
Wood 2012 [113]	Low	Low	Low	Low	Low	Low
Yamamoto 1995 [114]	Low	Low	Low	Low	Low	Low
Yanovski 2009 [123]	Low	Low	Low	Low	Low	Low
Zittermann 2009 [124]	Low	Low	Low	Low	Low	Low

Table S6: Results of sensitivity analyses for each supplement.

No. of Studies (S/D)	Baseline-End Corr	Cross-over Corr	Change in SBP I^2 (%)	Change in SBP MD (95% CI) CE	Change in SBP MD (95% CI) RE	Change in SBP PB	Change in DBP I^2 (%)	Change in DBP MD (95% CI) CE	Change in DBP MD (95% CI) RE	Change in DBP PB
**Calcium**										
23/23	0.7	-	25	-1.37 (-2.03, -0.71)	-1.54 (-2.35, -0.73)	N	66	-0.99 (-1.45, -0.53)	-1.63 (-2.70, -0.57)	Y
	0.5	-	8	-1.31 (-2.01, -0.61)	-1.41 (-2.20, -0.62)	N	54	-0.89 (-1.36, -0.41)	-1.50 (-2.45, -0.56)	Y
**Magnesium**										
18/18	0.7	0.9	95	-3.57 (-4.21, -2.93)	-2.78 (-5.22, -0.34)	N	93	1.00 (0.58, 1.43)	-1.63 (-3.11, -0.15)	Y
		0.7	95	-3.99 (-4.69, -3.29)	-2.79 (-5.25, -0.34)	N	92	1.31 (0.87, 1.75)	-1.56 (-3.03, -0.09)	Y
		0.5	95	-4.10 (-4.81, -3.38)	-2.81 (-5.28, -0.33)	N	91	1.40 (0.95, 1.84)	-1.51 (-2.98, -0.05)	Y
	0.5	0.9	95	-3.64 (-4.29, -2.98)	-2.76 (-5.31, -0.21)	N	92	1.22 (0.78, 1.65)	-1.51 (-3.02, 0.01)	Y
		0.7	95	-4.10 (-4.83, -3.38)	-2.77 (-5.34, -0.20)	N	90	1.56 (1.11, 2.02)	-1.42 (-2.92, 0.08)	Y
		0.5	95	-4.23 (-4.97, -3.48)	-2.78 (-5.37, -0.19)	N	90	1.66 (1.20, 2.12)	-1.36 (-2.85, 0.13)	Y
**Potassium**										
12/12	0.7	0.9	85	-1.05 (-1.52, -0.57)	-2.03 (-3.71, -0.36)	Y	82	-0.76 (-1.19, -0.33)	-1.25 (-2.51, 0.02)	N
		0.7	83	-1.61 (-2.22, -0.99)	-2.10 (-3.81, -0.38)	N	78	-1.13 (-1.65, -0.60)	-1.28 (-2.58, 0.02)	N
		0.5	83	-1.83 (-2.50, -1.16)	-2.15 (-3.90, -0.40)	N	76	-1.25 (-1.81, -0.70)	-1.31 (-2.64, 0.02)	N
	0.5	0.9	85	-1.05 (-1.53, -0.58)	-2.06 (-3.74, -0.37)	Y	82	-0.77 (-1.20, -0.34)	-1.27 (-2.55, 0.00)	N
		0.7	83	-1.62 (-2.24, -1.00)	-2.12 (-3.85, -0.39)	N	78	-1.15 (-1.67, -0.62)	-1.31 (-2.61, 0.00)	N
		0.5	82	-1.85 (-2.52, -1.18)	-2.18 (-3.94, -0.42)	N	76	-1.28 (-1.83, -0.72)	-1.34 (-2.68, 0.00)	N
**Vitamin C**										
5/4	0.7	0.9	23	-0.76 (-3.26, 1.75)	-1.12 (-4.15, 1.91)	N	0	-0.61 (-2.17, 0.94)	-0.61 (-2.17, 0.94)	N
		0.7	1	-1.45 (-4.26, 1.35)	-1.53 (-4.46, 1.40)	N	0	-0.47 (-2.29, 1.34)	-0.47 (-2.29, 1.34)	Y
		0.5	0	-1.64 (-4.52, 1.24)	-1.78 (-4.88, 1.32)	N	0	-0.43 (-2.32, 1.46)	-0.43 (-2.32, 1.46)	Y
	0.5	0.9	19	-0.72 (-3.57, 2.13)	-1.22 (-4.79, 2.36)	N	0	-0.69 (-2.44, 1.07)	-0.69 (-2.44, 1.07)	N
		0.7	0	-1.68 (-5.00, 1.64)	-1.73 (-5.19, 1.72)	N	0	-0.53 (-2.69, 1.63)	-0.53 (-2.69, 1.63)	N
		0.5	0	-1.96 (-5.41, 1.48)	-2.02 (-5.60, 1.56)	N	0	-0.47 (-2.76, 1.82)	-0.47 (-2.76, 1.82)	Y
**Vitamin D**										
29/28	0.7	-	15	-0.19 (-0.71, 0.33)	-0.21 (-0.80, 0.38)	N	53	0.17 (-0.18, 0.53)	0.11 (-0.47, 0.69)	N
	0.5	-	0	-0.06 (-0.62, 0.51)	-0.06 (-0.65, 0.53)	N	40	0.17 (-0.22, 0.56)	0.17 (-0.40, 0.73)	N
**Vitamin E**										
8/6	0.7	-	16	-1.76 (-3.05, -0.47)	-1.21 (-3.02, 0.6)	Y	0	1.17 (-0.51, 2.84)	1.17 (-0.51, 2.84)	N
	0.5	-	0	-1.76 (-3.42, -0.10)	-1.50 (-3.42, 0.42)	Y	0	1.17 (-0.99, 3.32)	1.17 (-0.99, 3.32)	N

Baseline-End Corr represents the correlation coefficient used for imputing missing standard deviations of change-from-baseline scores. Cross-over Corr represents the correlation coefficient used for calculating standard errors of mean differences. CE and RE represent common- and random-effects models. PB represents publication bias.

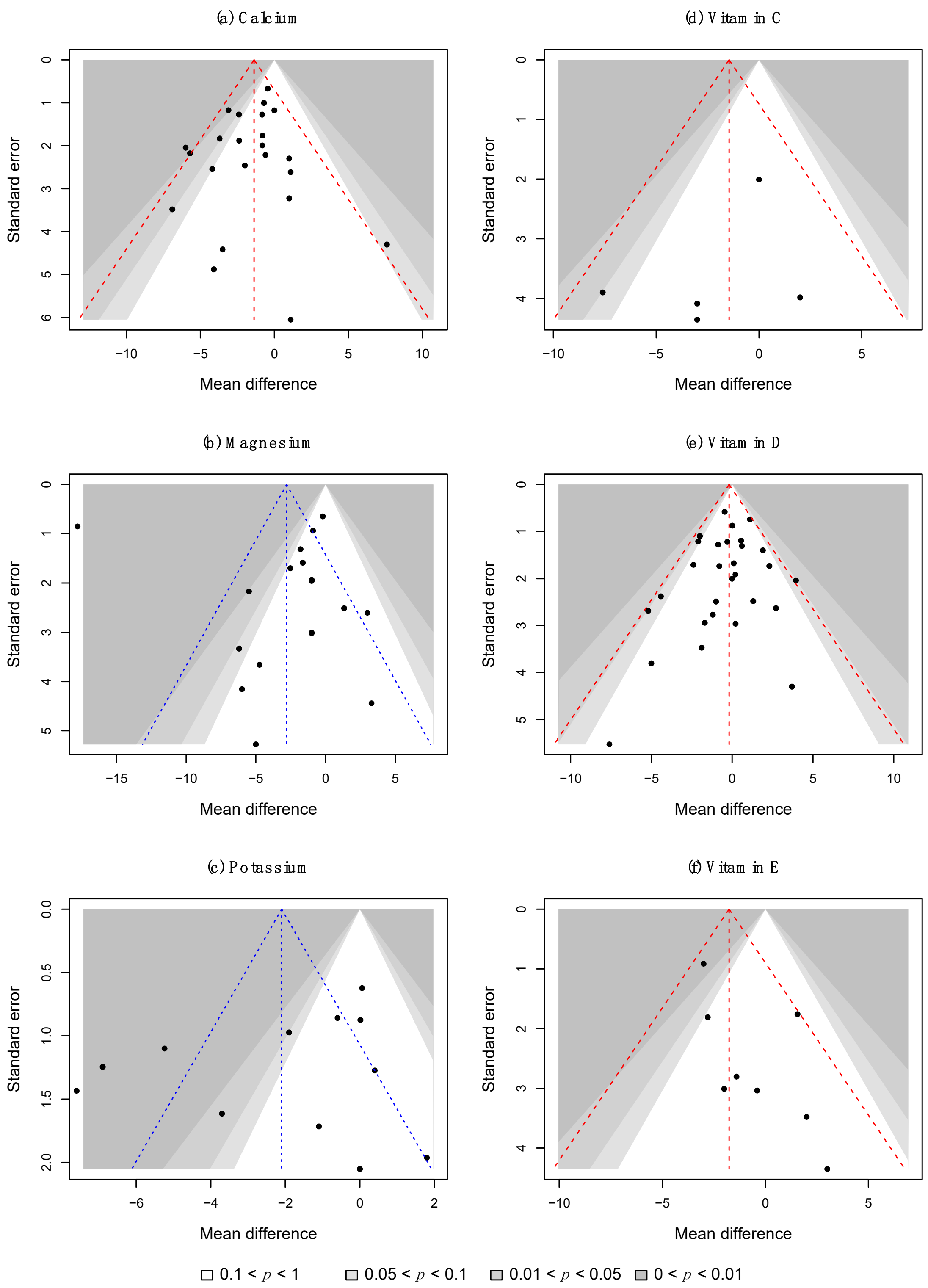

Figure S1: Contour-enhanced funnel plots of the six meta-analyses in systolic blood pressure reduction. The red or blue vertical and diagonal lines represent the overall MD and its 95% confidence limits based on the common-effect model or the random-effects model. Models are adopted due to heterogeneity. The shaded regions represent different significance levels for the effect size.

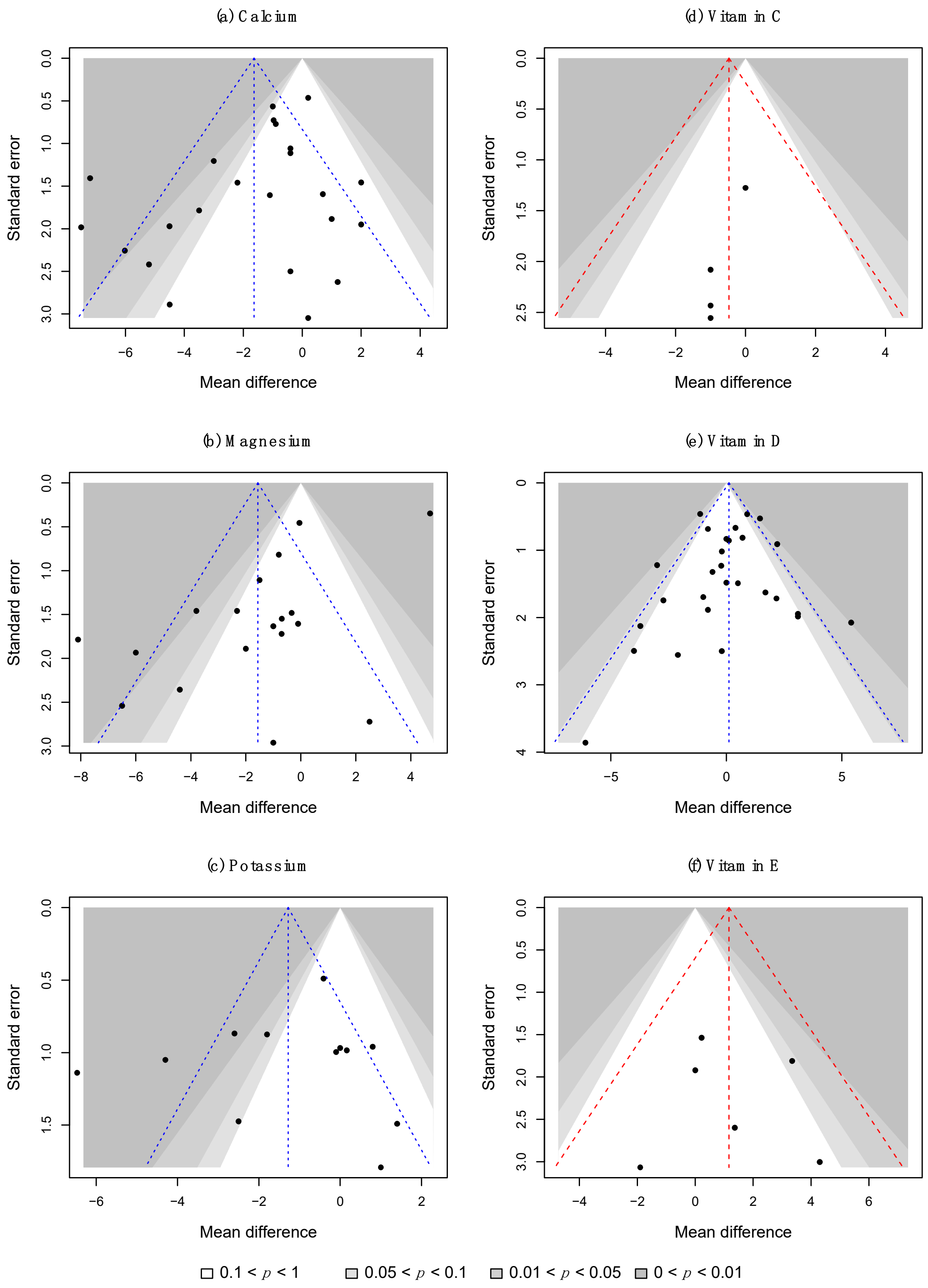

Figure S2: Contour-enhanced funnel plots of the six meta-analyses in diastolic blood pressure reduction. The red or blue vertical and diagonal lines represent the overall MD and its 95% confidence limits based on the common-effect model or the random-effects model. Models are adopted due to heterogeneity. The shaded regions represent different significance levels for the effect size.
